# In Vitro Characterization of Internalization Pathways and Cytotoxic Activity of Anti-HSPG2 Antibody–Drug Conjugates in MDA-MB-231-LM2 Cells

**DOI:** 10.3390/cancers18101638

**Published:** 2026-05-19

**Authors:** Zekun Shao, Lauren Morelli, Benjamin E. Blass, Andrey Efimov, Jayanth Panyam

**Affiliations:** 1 Department of Pharmaceutical Sciences, School of Pharmacy, Temple University, Philadelphia, PA 19140, USA; zekuns@uw.edu (Z.S.); lauren.morelli@temple.edu (L.M.); benjamin.blass@temple.edu (B.E.B.); 2 Department of Pharmaceutics, College of Pharmacy, University of Minnesota, Minneapolis, MN 55455, USA; 3 Department of Medicinal Chemistry, School of Pharmacy, University of Washington, Seattle, WA 98195, USA; 4 Fox Chase Comprehensive Cancer Institute, Temple University, Philadelphia, PA 19111, USA; andrey.efimov@fccc.edu; 5 Department of Pharmaceutics, College of Pharmacy, University of Washington, Seattle, WA 98195, USA

**Keywords:** ADC, linker chemistry, cellular internalization, cytotoxicity

## Abstract

Background/objectives: This study presents a mechanistic assessment of an anti-HSPG2 monoclonal antibody (AM6) as an antibody–drug conjugate (ADC) carrier in vitro. Methods: Using live-cell confocal imaging with pathway inhibitors, we qualitatively characterized AM6 internalization and trafficking and compared linker/payload configurations for intracellular delivery and in vitro cytotoxicity. Results: AM6 exhibited rapid cellular entry in MDA-MB-231-LM2 cells, with contributions from clathrin-mediated endocytosis and macropinocytosis, followed by accumulation in endo-lysosomal compartments. Consistent with these trafficking observations, AM6 ADCs bearing cleavable linkers and a potent payload (MMAE) produced more pronounced antiproliferative effects in MDA-MB-231-LM2 and other HSPG2-positive tumor cells than non-cleavable constructs, whereas doxorubicin-based ADCs showed limited activity and greater aggregation risk. Conclusions: Overall, the data inform linker/payload selection and highlight considerations for future work, including quantitative internalization, antigen-negative or knockdown controls, and in vivo pharmacology.

## Simple Summary

Antibody–drug conjugates are a promising type of targeted cancer therapy designed to deliver potent drugs directly to tumor cells while minimizing harm to healthy tissue. However, the effectiveness of a conjugate depends on how well it enters cancer cells and releases its drug payload inside them. In this study, we examine an antibody called AM6 that targets HSPG2, a protein commonly found on certain cancer cells, to better understand how cells take it up and how different drug-attachment strategies influence its activity. Using advanced imaging techniques, we tracked how AM6 moves inside cancer cells and tested several drug-linker designs. We found that certain drug combinations led to stronger cancer cell killing than others. These results provide practical guidance for designing more effective ADCs and highlight important considerations for future laboratory and preclinical studies in the field.

## 1. Introduction

Triple-negative breast cancer (TNBC) is a subtype of breast cancer lacking the expression of estrogen receptor (ER), progesterone receptor (PR), and the amplification of HER2 [1,2]. TNBC makes up 10–20% of total invasive breast cancer cases, which includes different molecular subtypes such as basal-like and claudin-low molecular subtypes. The TNBC subtype is characterized by a more malignant phenotype, shorter period to relapse, and poorer prognosis for patients compared to other breast cancer subtypes [2].

Until recently, due to the lack of specific breast cancer therapeutic targets such as HER2 and ER, the treatment of TNBC was largely reliant on traditional radiotherapy, chemotherapy, and surgery [2,3]. Radiotherapy and chemotherapy are associated with significant and dose-limiting toxicities, and most patients develop resistance to these therapies [4]. Thus, there is significant interest in developing targeted therapies to treat TNBC.

Antibody–drug conjugates (ADCs), a class of targeted chemotherapy consisting of an antibody conjugated to a cytotoxic payload through a linker, represent a novel form of targeted therapy for TNBC [3]. The antibody used in an ADC has a high affinity for cancer cells and can selectively deliver the conjugated payload to cancer tissue. Sacituzumab govitecan, an anti-Trop2 ADC, showed a significant improvement in overall survival in patients with metastatic TNBC compared with standard chemotherapy and is currently approved for patients with advanced TNBC who have previously received two or more lines of systemic therapy, including one in the metastatic setting. Trastuzumab deruxtecan, an anti-HER2 ADC, demonstrated anti-tumor efficacy against TNBCs previously considered HER2-negative, leading to the identification of a new HER2-low TNBC subtype [3,5].

Most ADCs utilize cellular internalization pathways for payload release [6]. Upon binding, the ADC is internalized by the cell and then transported into endocytic vesicles. ADCs delivered into endolysosomal vesicles are exposed to acidic, reductive, and enzyme-rich microenvironments. Under these conditions, payload release is contingent on full degradation of the antibody with non-cleavable linkers, or on rapid cleavage of the linker with cleavable linkers [6].

Various chemically reactive drug linkers have been developed to facilitate rapid payload release within the tumor [7]. The acid-labile hydrazone linker was used in the earliest approved ADC, gemtuzumab ozogamicin (Mylotarg^®^), which also employed a GSH-reducible disulfide moiety [8]. The disulfide linker was also used in the development of various maytansinoid ADCs. Steric hindrance around the disulfide bond impacted the in vivo efficacy [9]. The majority of clinically successful ADCs, including Adcetris [10] and Enhertu [11], employ a peptide linker that is cleaved by lysosomal enzymes, such as cathepsin B [12]. Other enzyme-cleavable linkers, such as β-glucuronidase-labile constructs [13], are also utilized in clinical ADC development.

As most ADCs rely on receptor-mediated endocytosis to achieve drug release, the internalization and specific endocytic pathways influence ADC drug release and, consequently, their anti-tumor efficacy. Although most ADCs enter cells via clathrin-mediated endocytosis (CME), other internalization pathways exist and may influence the pharmacological behavior of ADCs. For example, in the case of trastuzumab-emtansine (Kadcyla), the involvement of caveolae-mediated endocytosis (CAV) has been shown to induce drug resistance in HER2-positive cells [14]. The involvement of non-specific fluid-phase uptake led to non-specific ocular toxicity in vivo [15].

We previously reported the discovery of an anti-HSPG2 antibody (AM6) that binds to TNBC cells [16]. HSPG2 (perlecan) is a large, modular proteoglycan implicated in tumor biology [17]. Prior reports describing cell-surface HSPG2 in subsets of TNBC [16] motivated its examination here as a model antigen to study ADC trafficking and linker/payload performance in vitro. In the present work, we focus on the mechanistic internalization behavior of AM6 in a TNBC cell model (with validation in other HSPG2-positive tumor cell lines) and how linker chemistry and payload class influence intracellular delivery and in vitro cytotoxicity.

## 2. Methods

### 2.1. Cell Culture Materials

Unless otherwise specified, all the cell culture flasks and consumables were purchased from Genesee Scientific. The culture media (MEM and RPMI1640) and trypsin were purchased from Thermo Fisher (Waltham, MA, USA) and Fox Chase Cancer Center (Philadelphia, PA, USA).

### 2.2. Cell Culture

MDA-MB-231-LM2 cells (a lung metastasis derivative of the MDA-MB-231 cell line) were obtained from Dr. Joan Massague of the Howard Hughes Medical Institute, Memorial Sloan Kettering Cancer Center (New York, NY, USA). The cells were grown in MEM supplemented with 10% fetal bovine serum and 100 U/mL pen/strep (complete MEM) at 37 °C and 5% CO_2_. T24 cells and MB49 cells were obtained from ATCC. Both cell lines were grown in RPMI1640 supplemented with 10% FBS and 100 U/mL pen/strep at 37 °C and 5% CO_2_.

### 2.3. Antibody Production and Purification

AM6 was produced using the Expi293 expression system and purified using the protein A purification strategy. The plasmid was transfected per Thermo Fisher Expi 293 transfection protocol with V_L_: V_H_ = 2:1. On day 7, the filtered culture media were purified against Pierce™ Protein A Agarose beads (Cat # 20333; Thermo Fisher) in the column cartridge (bead volume = 0.5 mL) with Pierce™ Gentle Ag/Ab Binding/Elution Buffer (Cat# 21012 and 21027). The collected antibody elution was buffer exchanged into pH 8.0 Tris followed by dialysis into PBS. The protein concentration was measured using Nanodrop 2000 (Thermo Fisher, Waltham, MA, USA).

### 2.4. Synthesis of Doxorubicin Linkers

#### 2.4.1. PDPH-Doxorubicin (CAS# 131176-38-0)

A total of 75 mg Doxorubicin HCl (CAS# 25316-40-9) and 60 mg (3-(2-pyridyldithio)propionyl hydrazide) (PDPH, CAS# 115616-51-8) and catalytic TFA were dissolved together in MeOH and stirred for 5 days. The solvent was then evaporated at 28 °C. The red solid was washed with acetonitrile. The solid product was dissolved with MeOH and concentrated, then purified on a Gemini 5 μm C18 column using an acetonitrile gradient from 10 to 100% in water. The final product (20.5%) was confirmed with LC-MS with *m*/*z* [M + H] + 757.2 and purity > 98% (Figure S1a).

#### 2.4.2. Maleimidocaproyl-Doxorubicin (Mc-Dox, MC# 290239)

A total of 50 mg Doxorubicin HCl (CAS# 25316-40-9), 33 mg 6-maleimidohexanoic acid N-hydroxysuccinimide ester (mc-NHS, CAS# 55750-63-5), and 25.8 mg DIPEA were dissolved in 1.5 mL DMF and stirred overnight. The solvent-evaporated products were redissolved in 3.5 mL MeOH and filtered. Then, the product was purified on a C18 column using acetonitrile 10–100%/water gradient with 1% formic acid. The product was evaporated and mixed with 2 ml diethyl ether. The final product (31.5%) was confirmed with LC-MS with *m*/*z* [M + Na]^+^ 759.2 and a purity > 98% (Figure S1b).

#### 2.4.3. SMCC-Doxorubicin (SMCC-Dox, CAS# 400647-59-8)

The SMCC-dox was directly purchased from MedChemExpress (Cat. No.: HY-116063; Monmouth Junction, NJ, USA).

#### 2.4.4. PDP-Doxorubicin (MC# 310008)

To a solution of doxorubicin-HCl (30 mg, 0.05 mmol, CAS# 25316-40-9) dissolved in DMF (1.0 mL), DIPEA (13 mg, 0.1 mmol) was added dropwise. A solution of succinimidyl 3-(2-pyridyldithio) propionate (SPDP) (23 mg, 0.075 mmol) in DMF (1.0 mL) was then added to the reaction mixture. The reaction mixture was stirred for 12 h at room temperature. DMF was then removed under reduced pressure. The crude product was purified on a silica gel column (0–10% MeOH/DCM), resulting in a final product which was a dark red oil (54%), *m*/*z* [M + H]^+^ 741.17 (Figure S1c).

#### 2.4.5. Mc-Val-Cit-Doxorubicin (Vc-Dox, CAS# 159857-70-2)

Doxorubicin-HCl (15 mg, 23 µmol) and mc-Val-Cit-PABC-PNP (17.5 mg, 23 µmol) linker were suspended in anhydrous DMF (1.5 mL). DIPEA (2.1 µL) was added to the above reaction mixture dropwise, resulting in a homogenous solution. The reaction mixture was stirred for 12 h at room temperature. DMF was then removed under reduced pressure. The crude product was purified on a silica gel column (0–20% MeOH/DCM), resulting in a red solid final product (62%). The product was confirmed by mass spectrometry, showing a peak at *m*/*z* [M + Na]^+^ 1164.71 (Figure S1d,e).

#### 2.4.6. Doxorubicin-Linker Fluorescence Measurements

Doxorubicin-linker conjugates were dissolved in DMSO to produce a stock solution at 5–10 mg/mL concentration. Serial dilutions were performed in 10% DMSO-supplemented ddH_2_O to avoid the salting-out effect. A total of 30 µL of the diluted solutions was transferred to a 384-well plate. Fluorescence of the solutions was measured using the SpectraMax i3x microplate reader with excitation at 490 nm and emission at 590 nm.

### 2.5. Antibody–Drug/Fluorophore Conjugation

The doxorubicin-linker synthesis was described in the previous section. All the MMAE linkers were purchased from MedChemExpress: mc-val-cit-MMAE (CAS# 646502-53-6, cat# HY-15575), mc-MMAE (CAS# 863971-24-8, cat# HY-15741) and MC-beta glucuronide-MMAE (CAS# 1703778-92-0, cat# HY-136317).

The antibody–drug conjugation was performed using an interchain-disulfide reduction-conjugation approach with maleimide-thiol click chemistry using Tris(2-carboxyethyl)phosphine hydrochloride (TCEP·HCl) as the reductant. Specifically, antibodies in PBS were treated with DTPA and a phosphate-based pH-adjustment buffer to create a favorable environment for TCEP reduction. Then, based on the desired DAR, a 2*DAR molar equivalent of TCEP was added to the antibody and incubated at 37 °C for 1 h. The linker–payload, dissolved in DMSO, was added directly to the reaction mixture for conjugation at RT for 45 min. The antibody–drug/fluorophore conjugates were then purified and collected using a 7k Zeba Spin column. The concentrations of ADC products were measured using a Nanodrop 2000 UV-Vis Spectrophotometer.

### 2.6. Characterization of the Conjugates

#### 2.6.1. SDS-PAGE

Bio-Rad 4–15% Criterion™ Tris-HCl Protein Gel (Cat # 3450027; Bio-Rad, Hercules, CA, USA) was used for SDS-PAGE analysis. For non-denaturing studies, the protein samples were mixed 1:1 with the loading buffer and directly loaded for SDS-PAGE. For denaturing studies, the protein samples were mixed 1:1 with the loading buffer and 0.5 M DTT to produce a final concentration of 50 mM DTT. The samples were then heated in a heating block at 90 °C for 30 min. The electrophoresis settings were 150 V, 1 h in 1× Tris-glycine buffer.

#### 2.6.2. Hydrophobic Interaction Chromatography (HIC)

The HIC analysis of antibody-MMAE ADCs was performed on an Agilent 1260 Infinity II LC System with a bioinert pump using a TOSOH TSKgel Butyl-NPR column (4.6 mm × 3.5 cm, P/N 0014947). The column was equilibrated with 75% high-salt binding buffer A (2 M ammonium sulfate, 50 mM sodium phosphate, pH 7) and 25% low-salt elution buffer B (50 mM sodium phosphate, pH 7) for 6 min. A gradient of 75–0% buffer A, 25–75% buffer B, and 0–25% IPA was applied over 20 min under RT. The 75% buffer B–25% IPA wash step was maintained for 5 min; the 100% buffer B was run for 3 min before switching to high-salt buffer to avoid ammonium sulfate salt-out. The flow rate was maintained constant at 0.5 ml/min. For each run, 14 µL of ADCs (~2 mg/mL) were mixed with 21 µL buffer A for column binding. A total of 30 µL of mixed samples were injected. The signal was acquired at 215, 227, 254, 280, 490 and 647 nm wavelengths simultaneously. The signal analysis was performed using Agilent Openlab CDS. Unless otherwise specified, the HPLC system used, the signal acquisition, and analysis were unchanged for other LC methods described below.

#### 2.6.3. Size-Exclusion Chromatography (SEC)

The SEC analysis of antibody and ADCs was performed using an Agilent AdvanceBio SEC column (200 Å 1.9 um 4.6 × 300 mm, Part No. PL1580-5201) connected to a guard column (PL1310-0005). A mobile phase consisting of 150 mM sodium phosphate buffer, pH 7, was run in isocratic mode at 0.28 mL/min for 20 min at RT.

#### 2.6.4. PLRP-qTOF

The PLRP-qTOF analysis was performed at Frontage Labs, Exton, PA. The acquisition of LC-MS signal was performed using the Sciex TripleTOF 6600 LC-MS System. The Agilent PLRP-S chromatographic column, 2.1 × 50 mm, 5 μm, 1000 Å (PN: PL 1912-1502) was used. The LC gradient and MS acquisition methods were adopted from Agilent application note 5991-6559EN. Chromatogram analysis was performed using Ab Sciex Peakview Version 2.1 software.

### 2.7. Confocal Imaging

#### 2.7.1. Cell Seeding

About 10^4^ LM2 cells were seeded in 1.5 mL of complete MEM on a MATTEK 35 mm glass bottom confocal dish (part # P35G-1.5-14-C) two days before imaging to ensure cell adherence and normal morphology.

#### 2.7.2. Treatments

For the endocytosis inhibition study, the following inhibitors were applied to the cells: 10 μg/mL chlorpromazine HCl (Cayman chemicals, CAS No. 69-09-0, cat # 16129) for CME inhibition, 54 μg/mL genistein (Cayman chemicals, CAS No. 446-72-0, cat # 10005167) for CAV inhibition, and 30 μg/mL EIPA (Cayman chemicals, CAS No. 1154-25-2, cat # 14406) for macropinocytosis inhibition. These inhibitor doses were tested for cytotoxicity using MTS assay (see the cell viability section for details). Markers for various endocytic pathways were as follows: transferrin-CF488A (Biotium Cat# 00081) for CME, cholera toxin subunit B-CF488A (Biotium Cat # 00070) for CAV, and dextran 70-Oregon Green 488 (Thermo Fisher D7172) for macropinocytosis. All the inhibitors were dissolved in complete MEM and prewarmed to 37 °C for 15 min before use. The inhibitor-supplemented media were applied to the cells for 30 min prior to the antibody treatment.

To track antibody internalization, 50 nM Alexa Fluor 647-labeled AM6 or Cetuximab (see below for conjugation procedure) in complete MEM was applied to cells, which were then observed directly under a confocal microscope. For doxorubicin ADC release studies, each of the doxorubicin-antibody conjugates (see below for conjugation process) was diluted to 1 μM antibody concentration for DAR 4 ADCs or 400 nM for DAR 8 ADCs using complete MEM. The diluted treatments were then applied to the cells for overnight (~16–18 h) incubation under cell culture conditions. Before the imaging procedure, a 1:80,000 dilution of 10 mg/mL Hoechst 33342 (Thermo Fisher) was added to the treatment media. The imaging dish was equilibrated in the incubator for 15 min before confocal observation.

#### 2.7.3. Imaging Procedure

Imaging was conducted on a Leica SP8 confocal microscope at Fox Chase Cancer Center. The humidified imaging chamber for live cell imaging was set to 37 °C and 5% CO_2_. All the images are taken using a 40× objective lens. A 633 nm laser at 5% intensity was used to observe the AF647-labeled antibody. A 1 μm step z-stack image series was acquired every 20 min, starting at 10 min and continuing until the next day (16–18 h). For the intracellular doxorubicin release study, a 405 nm laser at 5% intensity was used for Hoechst 33342 imaging, and a 514 nm laser at 5% intensity was used to track doxorubicin-antibody conjugates. A 1 μm step z-stack image series was acquired at the end point of treatment (26–28 h). All the confocal picture analyses (pixel volume, Mander’s coefficient for colocalization study, z-stacking and video piling) were performed with FIJI.

### 2.8. Cytotoxicity Studies

MDA-MB-231-LM2 cells (2000 cells in 50 µL media) were seeded in 96-well plates and allowed to attach overnight. ADC treatments were prepared in 50 µL of media to achieve twice the desired final concentrations, then serially diluted. Various concentrations of the ADCs were then added to the wells containing media to achieve a 1:1 dilution. The plate was incubated for 72 h before cell viability testing.

Cell viability was assessed using CellTiter-Glo and MTS (both from Promega, Madison, WI, USA). For CellTiter-Glo assay, the Promega CellTiter-Glo^®^ Luminescent Cell Viability Assay Kit (Ref #: G7572) was used per protocol. The kit buffer and substrate components were mixed and then added directly (100 μL per well of a 96-well plate) to the cells. After shaking at 600 rpm for 30 min, the luminescence data were collected with a Spectra i3 Max plate reader.

Amounts of 2 mg/mL MTS and 0.92 mg/mL PMS, adjusted to pH 7.4, were prepared for the MTS assay. For the assay, 20 µL of 1:20 PMS:MTS premix was added to the cells in the treatment medium and incubated at 37 °C for 3 h or until sufficient absorbance was reached. The absorbance at 490 nm was determined with a Spectra i3 Max plate reader.

## 3. Results

### 3.1. Internalization of AM6

#### 3.1.1. Pulse-and-Chase Tracking of AM6 Antibody Internalization

**Figure 1:**
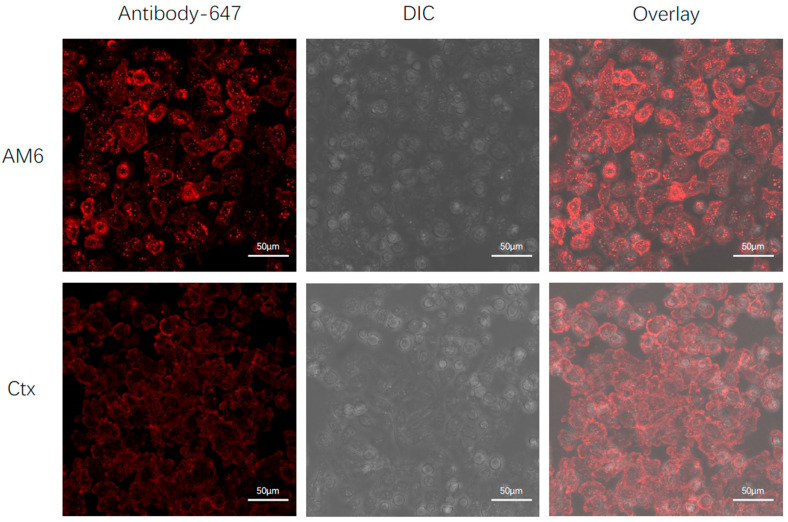
Confocal images of AM6 and Ctx internalization by LM2 cells at 1 h timepoint. The red channel indicates the AF647-labeled antibody signal. DIC—differential interference contrast. Images are shown as z-stack.

The intracellular location and extent of ADC payload release are dictated by antibody internalization and subcellular localization. Thus, to select a suitable linker that cleaves under specific subcellular microenvironments, we evaluated the antibody intracellular trafficking. The LM2 cell line was treated with Alexa Fluor 647-labeled antibody and then imaged using live-cell confocal microscopy. Figure 1 shows the 1 h timepoint slice for the image series. The 10 min movie series is attached in Figure S2. We noticed photobleaching of the imaging area at later time points (>8 h). Nonetheless, the image series at earlier time points indicates punctate fluorescence within the cells for AM6, suggesting that AM6 is in intracellular vesicles by one hour. At the same time, Cetuximab (Ctx) remained mostly surface-bound for up to 90 min, suggesting that AM6 undergoes much quicker internalization than Ctx in the LM2 cell line. These observations are qualitative and, while consistent with more rapid AM6 entry in LM2 than Cetuximab, do not provide quantitative internalization rates or establish antigen dependence.

#### 3.1.2. Characterizing the Effect of Endocytic Inhibitors on Antibody Internalization

The internalization pathway of a ligand dictates its intracellular localization and biotransformation. CME is a classic endocytic pathway in which the clathrin-coated vesicles engulf the ligand and efficiently transport it toward the endo-lysosome, where the ligand degrades under an acidic and enzyme-rich environment. Meanwhile, other endocytic pathways, such as caveolae-mediated endocytosis (CAV) [18], and pinocytotic pathways, such as macropinocytosis (MPC), may lead to alternative intracellular distribution and metabolic profiles of the ligands [19]. We utilized individual endocytic inhibitors to determine the involvement of the three endocytic pathways of interest in AM6 and Ctx internalization. MTS assay and endocytic marker pulse-and-chase imaging (transferrin-CF488A for CME, cholera toxin subunit B-CF488A for CAV, and dextran 70-Oregon Green 488 for MPC) experiments were first performed to confirm minimal effects on cell viability (Figure S3) and pathway inhibition (Figure S4) with these inhibitor treatments. The cells were exposed to inhibitor-supplemented media for each inhibitor group prior to treatment with the inhibitor-antibody combination. The image series was then immediately captured every 20 min.

**Figure 2:**
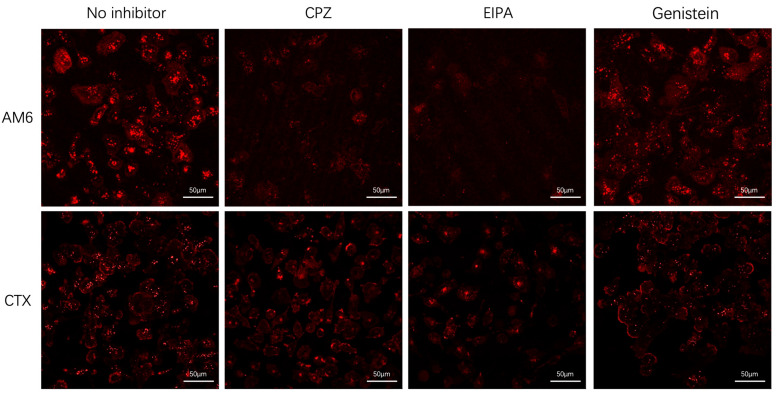
Confocal imaging of AM6 and Ctx internalization at the 2 h timepoint by LM2 cells with or without individual endocytic inhibitors. Inhibitor and the corresponding pathway: chlorpromazine-CME; CAV-Genistein; macropinocytosis-EIPA. Images are shown in z-stack.

We observed that internalization of both antibodies was inhibited by the CME inhibitor chlorpromazine (CPZ) and the macropinocytosis inhibitor 5-(N-ethyl-N-isopropyl) amiloride (EIPA). The 2 h timepoint images are shown in Figure 2. While Ctx internalization was more susceptible to CPZ inhibition, AM6 internalization was greatly affected by EIPA inhibition. The CAV inhibitor genistein did not significantly affect the internalization of either antibody. Overall, the results from this study showed that (1) both antibodies utilize CME and (2) internalization of AM6, but not of Ctx, involves macropinocytosis.

### 3.2. Investigating the Effect of the Linker Used on Intracellular Doxorubicin Drug Release

#### Conjugation of Doxorubicin-Linkers to Antibodies

ADC drug release is contingent on cellular uptake of the ADC and on the reactivity of the drug-linker within specific subcellular microenvironments. Different cleavable linkers, such as endo-lysosomal cleavable peptides and glucuronide linkers, have been utilized in commercial ADC products to facilitate payload release in vitro and in vivo [20]. Building on insights into the internalization and endocytic pathways of the AM6 and Ctx antibodies from the previous study, we investigated the impact of various linkers on intracellular drug release from AM6 and Ctx ADCs. We selected doxorubicin because of its established, facile chemistry for synthesizing various linker conjugates and its natural fluorescence, which enables confocal-based tracking of intracellular drug release.

**Figure 3:**
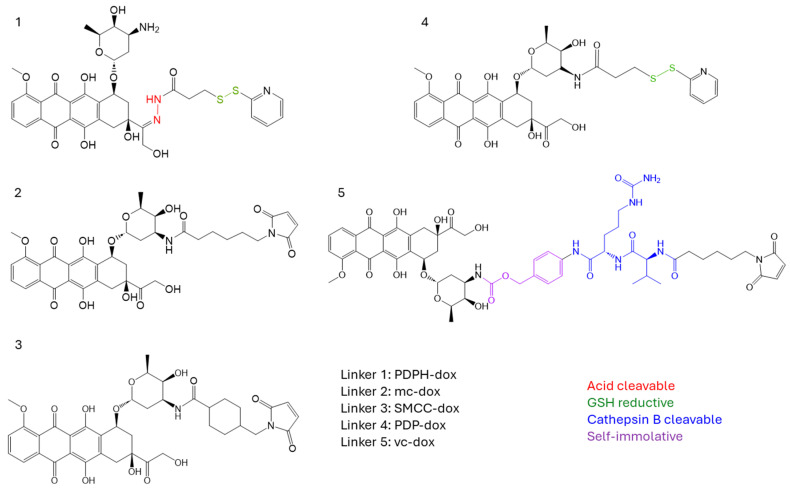
Chemical structures of the five dox-linkers used in the study.

**Figure 4:**
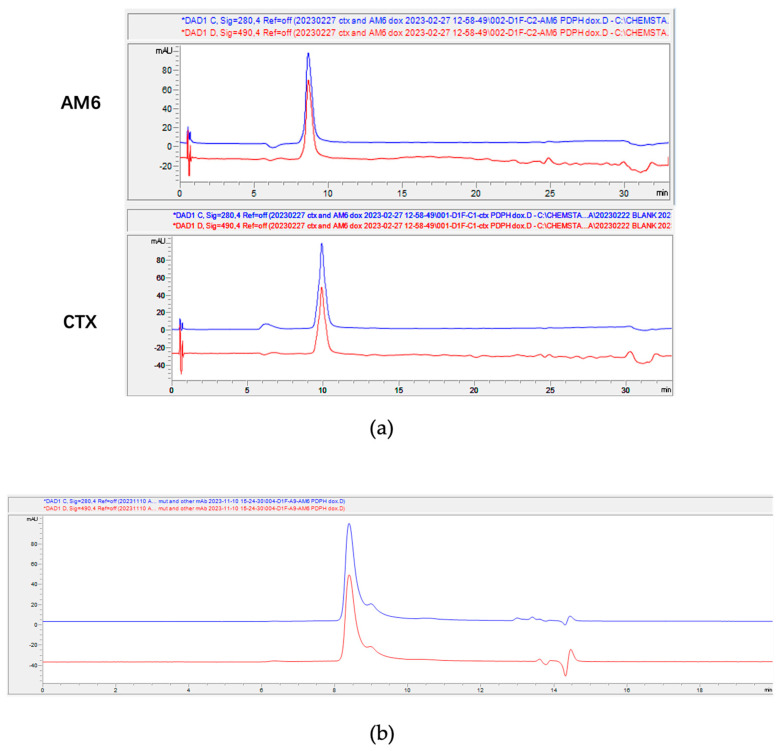
(**a**) HIC analysis of antibody doxorubicin-PDPH conjugate. Blue curve: 280 nm absorbance corresponding to the protein signal. Red curve: 490 nm absorbance corresponding to the doxorubicin signal. (**b**) SEC analysis of AM6 doxorubicin-PDPH conjugate. Blue curve: 280 nm absorbance corresponding to the protein signal. Red curve: 490 nm absorbance corresponding to the doxorubicin signal.

We selected five different doxorubicin-linker moieties that were either commercially available or could be readily synthesized. The structure of the drug linkers is shown in Figure 3. The hydrazide moieties in dox-linker 1 can be cleaved under an acidic environment. The disulfide bonds in PDPH linker (1) and PDP linker (4) are cleavable by intracellular reductive GSH. The mc-val-cit-PAB linker (5) is cathepsin cleavable and has been extensively used in commercial ADCs. The mc linker (2) and SMCC linker (3) are non-cleavable. The SMCC linker is more hydrophobic than the mc linker. For the compounds synthesized in-house, we confirmed their molecular weight by LC-MS (Figure S1a–e). Conjugation of each of the doxorubicin-linkers to the TCEP-reduced antibody was confirmed by both HIC (Figure 4a and Figure S5) and SEC (Figure 4b and Figure S6). Due to the limited amounts of the synthesized compounds and low fluorescence levels, it was difficult to establish standard curves for each drug-linker conjugate, thereby limiting accurate quantification of the drug-to-antibody ratio (DAR) for these doxorubicin ADCs using colorimetric approaches. Furthermore, HIC does not separate and identify the mAb-dox conjugate subspecies (DAR = 0, 2, 4, 6, 8) like it can for MMAE-mAb conjugates because of the low hydrophobicity of doxorubicin payloads (Figure 4a and Figure S5). Because of this, we were limited to calculating the DAR based on the assumption that moles of doxorubicin added to the antibody was approximately twice the moles of TCEP used. We also measured the fluorescence at 590 nm per proximal unit of these dox-linkers (Table S1). Given the comparable 590 nm fluorescence per unit of dox-linker and the same TCEP reduction level for ADC conjugation, together with the confocal microscopy result in the next section, the differences in DAR, if any, of these dox-mAb conjugates did not affect the conclusion from the intracellular doxorubicin release study.

### 3.3. Characterizing the Intracellular Release of Doxorubicin ADC with Confocal Microscopy

**Figure 5:**
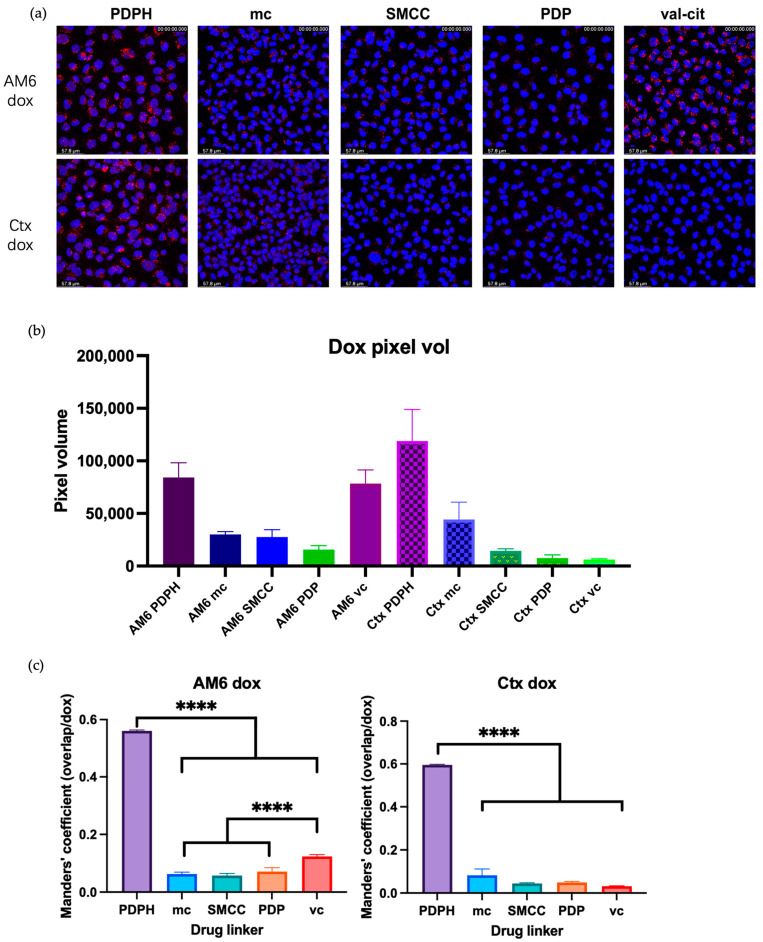
Doxorubicin ADCs with different linkers were tested for their intracellular drug release. (**a**) Images with z-slices were taken 26–28 h post antibody incubation. Images are shown in z-stack. (**b**,**c**) Quantified analysis of the images in Figure 5a; (**b**) quantification of the doxorubicin 514 nm channel pixel volume; (**c**) quantification of doxorubicin red pixel colocalizing with the blue Hoechst nucleus signal (*n* = 3, **** *p* < 0.0001).

We next characterized the intracellular release of doxorubicin from the ADCs in LM2 cells using live-cell-based confocal microscopy. When released inside the cell, free dox can diffuse into the nucleus and intercalate with genomic DNA. Thus, nuclear localization of dox was considered as a marker for intracellular release from the ADC. The nucleus was stained using Hoechst 33342. The z-stacked images are shown in Figure 5a. The Mander’s coefficient and quantification of the red pixels for doxorubicin were analyzed and are shown in Figure 5b,c.

These images (Figure 5a) and analysis results (Figure 5b,c) show that the use of PDPH-dox linker results in intracellular doxorubicin release with both AM6 and Ctx ADCs. The other linker-dox candidates did not achieve a similar level of intranuclear doxorubicin delivery. However, the val-cit-dox, when conjugated to AM6 but not to Ctx, resulted in the accumulation of doxorubicin in intracellular vesicles.

While this study revealed differences in intracellular drug release among linkers, all the doxorubicin ADCs failed to induce cytotoxicity in LM2 cells, even at high doses. MTS assay results further suggested that doxorubicin may not be a potent ADC payload, with the IC50 reaching approximately 200 nM. The IC50 data from GDSC datasets of doxorubicin on the parent cell line MDA-MB-231 further confirmed this observation (Figure S7).

Additionally, some doxorubicin conjugates formed aggregates upon storage. The presence of aggregates was confirmed using SEC (Figure S6). Given the insufficient potency and the tendency to aggregate, we shifted our focus from doxorubicin to MMAE, a more potent and stable payload for further studies.

### 3.4. Conjugation of MMAE

**Figure 6:**
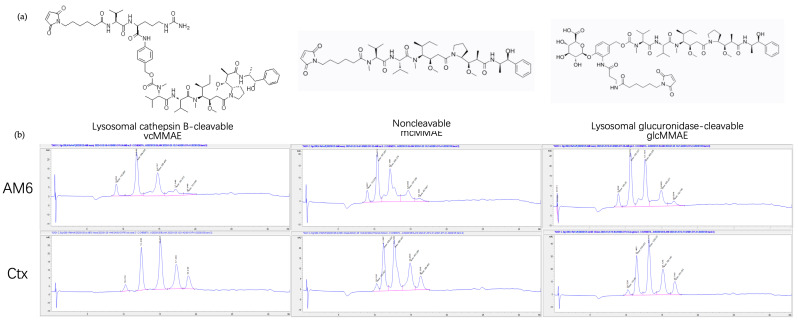
Antibody-MMAE conjugation and HIC characterization. (**a**) Chemical structure of val-cit-MMAE, mc-MMAE and mc-β-glucoronide-MMAE. (**b**) HIC results of the different antibody-MMAE conjugates.

Of the various payloads used in commercial ADCs, we chose MMAE based on the following considerations: (1) MMAE has been broadly tested for its potency on cells used for multiple commercial ADC products; (2) MMAE payloads with various linkers are commercially available; (3) analytical methods such as HIC are available for DAR analysis. We selected mc-val-cit-PAB-MMAE and mc-β-glucuronide-MMAE for creating model lysosomal cleavable ADCs and mc-MMAE for creating a non-cleavable ADC (Figure 6a). These MMAE conjugates were characterized by HIC (Figure 6b) and PLRP-qTOF (Figure S8). Based on HIC, the average DARs for these ADCs were around 3.6–4.0.

### 3.5. In Vitro Cytotoxicity

**Figure 7:**
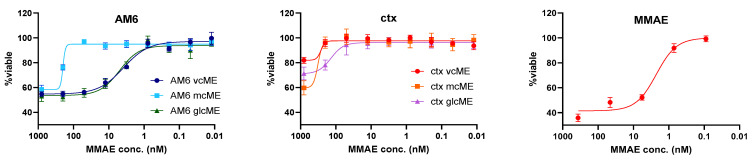
Cytotoxicity of various antibody-MMAE conjugates against LM2 cells at 72 h, *n* = 4 for each point.

We next characterized the cytotoxicity of the MMAE ADCs against LM2 cells. Figure 7 shows the efficacy of these ADCs after 72 h of exposure. AM6 ADCs were more effective than Ctx ADCs. While all three AM6 ADCs showed toxicity against LM2 cells, we observed that using lysosomal-cleavable linkers resulted in the greatest efficacy of AM6 ADCs. We confirmed the cytotoxicity of these ADCs against other HSPG2-positive, EGFR-negative cell lines (Figure S9). While Ctx ADCs were inactive in Ctx-negative cells, consistent with receptor expression, we did not include HSPG2-negative controls for AM6 in this study; therefore, the contribution of antigen-dependent delivery to AM6-ADC cytotoxicity cannot be conclusively established. In these assays, some dose–response curves did not reach a classical IC50 within the tested concentration range; as such, results are interpreted comparatively rather than as potency benchmarks.

## 4. Discussion

The current study investigated the correlation between antibody internalization, intracellular localization, and the drug-release profile of AM6- and Ctx-based ADCs using different linkers. The internalization rate and endocytic pathways have previously been shown to affect ADC performance [21]. The clinical ineffectiveness of the CD33 ADC gemtuzumab ozogamicin has been attributed to the slow internalization of CD33 via CME [21]. Meanwhile, the major involvement of CAV in HER2 internalization was attributed to the development of resistance to trastuzumab-emtansine [14].

Prior reports have identified cell-surface expression of HSPG2 in subsets of TNBC clinical specimens and model systems [16], motivating its exploration as a molecular handle for intracellular delivery rather than as a validated therapeutic vulnerability. Publicly available resources and histopathologic summaries further indicate that perlecan is a large, widely distributed extracellular matrix proteoglycan with known roles in normal tissue architecture (https://www.proteinatlas.org/ENSG00000142798-HSPG2, accessed 17 May 2026), underscoring the need for careful consideration of on-target exposure beyond tumor cells. In the present study, HSPG2 was examined solely as a model antigen to probe antibody internalization, trafficking, and linker/payload behavior in vitro. Safety, normal tissue expression-driven liabilities, and therapeutic index were not evaluated and cannot be inferred from the data presented.

HSPG2 has been reported to mediate the cellular internalization of various biochemical moieties, including exosomes and growth factors such as fibroblast growth factor 2 (FGF2) [22,23]. Although existing research links HSPG2-induced internalization to MPC [24], HSPG2 internalization remains poorly characterized. As observed in our internalization studies, HSPG2 was internalized rapidly via MPC, and this internalization was inhibited by the MPC inhibitor EIPA. We also observed inhibition by the CME inhibitor chlorpromazine, suggesting a possible role for CME in AM6 internalization. It is worth noting that MPC is a recognized, target-independent route for ADC uptake [25]. It should be noted that the fluorescence signal observed in our studies indicates the location of AM6, not HSPG2, because the interaction between AM6 and HSPG2 under macropinosome/lysosomal conditions has not been characterized. It is possible that AM6 dissociates from HSPG2 at some point during internalization.

We used Ctx as a benchmark control. Cetuximab binds to the epidermal growth factor receptor (EGFR) which is expressed in various cancer cell lines, including the MDA-MB-231 cell line used in our study. The internalization of Ctx has been extensively studied because of its clinical relevance. In addition to the classic CME pathways, clathrin-independent pathways, such as MPC, have been shown to play a vital role in modulating EGFR downregulation in cancer cells [26]. Our imaging data confirmed these previous observations and indicated slower internalization and lysosomal accumulation of Ctx compared to AM6, which may contribute to the differences observed between Ctx- and AM6-based MMAE constructs in this cell model, although additional controls would be required to isolate the mechanisms.

In our initial studies to characterize different drug linkers, we chose doxorubicin as the model payload due to its facile reaction with various drug linkers we are interested in. These doxorubicin-linker structures and their synthesis routes are well established [27]. Among these combinations of antibodies and linker-doxorubicin moieties, PDPH-dox led to nuclear delivery of doxorubicin with both AM6 and Ctx. However, this does not necessarily suggest that the PDPH linker is superior to the other linkers. The major concern with the PDPH linker is instability. Our initial work evaluating PDPH-dox release showed rapid doxorubicin release under acidic pH (pH 4.5 and pH 5.0). However, previous studies show that ~10% of doxorubicin was released from the drug-linker complex even at pH 7.4 [27]. Additionally, we observed a small but visible dark red precipitate in the AM6 and Ctx PDPH-dox solutions, but no significant aggregate peaks were observed in the SEC results (Figure S6). Combining these facts, we speculate that doxorubicin released from the PDPH-dox mAb and localized in the nucleus could result from extracellular hydrazone cleavage. Further, the PDPH-linked conjugate-treated cells displayed a distinct nuclear appearance under microscopy. However, there is no prior evidence that PDPH linkers directly induce nuclear swelling or stress-associated morphological changes; nevertheless, we cannot exclude the possibility that subtle linker-dependent cellular stress effects may influence intracellular morphology or uptake behavior, and the observed localization should therefore be interpreted as correlative rather than definitive evidence of enhanced internalization. While the other dox-linkers failed to achieve nuclear delivery of doxorubicin, the val-cit ADC resulted in lysosomal accumulation in AM6 but not in Ctx. This further supports the lysosomal accumulation of AM6 observed in the pulse-chase imaging studies (Figure 1 and Figure 2). The sequestration of doxorubicin in the vesicle also suggests possible protonation of the released doxorubicin [28]. A caveat here is that aggregation can sequester higher-DAR species, thereby lowering the apparent DAR in the soluble fraction.

In our SEC characterization of these dox ADCs, we observed varying degrees of aggregation, except for PDPH-dox. We propose two possible explanations for this phenomenon. First, the aggregation is covalent and caused by the nucleophilic attack of the ketone carbon on the doxorubicin α-hydroxyl ketone by the free thiol group. Hydrazone formation may reduce the electron-withdrawing effect of PDPH, thereby reducing aggregation in PDPH-dox ADCs. Second, the aggregation is non-covalent and arises from hydrophobic interactions between linker-doxorubicin payloads. SMCC-dox and val-cit dox ADCs are more hydrophobic, resulting in greater aggregation. PDPH-dox is likely less hydrophobic because of the charge on the hydrazone and free amino groups in doxorubicin. Previous work reported the aggregation of high-DAR PDPH-dox ADCs [29]. That study did not detect crosslinking by denaturing gel electrophoresis, which supports the possibility of non-covalent aggregation. We performed SDS-PAGE under both normal and reducing conditions to further characterize the aggregates (Figure S10). Multiple >50 kDa bands persisted after reductive (50 mM DTT) and denaturing (SDS + 90 °C) conditions, indicating the crosslinking of chains in the case of SMCC-dox and val-cit-dox.

In the efficacy study, we observed greater cytotoxicity with AM6 ADCs than with Ctx ADCs, likely due to their faster internalization. While the greater efficacy of cleavable MMAE ADCs compared to non-cleavable MMAE ADCs was expected, we did not see a significant difference in efficacy between val-cit and glucuronide-MMAE AM6 ADCs. The comparison of these two linkers has been investigated with payloads such as camptothecin and MMAE [30,31]. A previous study investigated three different antibodies with glucuronide and val-cit linkers. Compared with the highly aggregation-prone val-cit linker ADCs, glucuronide ADCs exhibit a lower tendency to aggregate, primarily due to their hydrophilicity. In CD70 ADCs, where both linkers resulted in <5% aggregation, the glucuronide-linker ADCs were more efficacious than the val-cit-linker ADCs in mouse xenograft models [24]. However, another study by the same group observed no differences in the in vivo efficacy of CD30-targeted ADCs comprising either glucuronide- or val-cit-MMAE [31].

## 5. Conclusions and Future Studies

In the current study, we conducted a mechanistic, in vitro evaluation of AM6 as an ADC carrier in a TNBC cell model. Our confocal imaging suggests that AM6 enters LM2 via clathrin-mediated endocytosis, with a contribution from macropinocytosis, resulting in endolysosomal accumulation. Consistent with this trafficking, AM6 ADCs bearing lysosomal-cleavable linkers and MMAE showed more pronounced antiproliferative effects than non-cleavable constructs, whereas doxorubicin-based ADCs showed limited activity and a higher tendency to aggregate. These results inform linker/payload selection at an early stage of ADC design.

## 6. Study Scope and Limitations

The conclusions of this work should be interpreted within the defined experimental scope. Established ADC targets in TNBC, such as Trop-2 and HER2-low, are cited here for contextual grounding rather than comparison, as their clinical validation reflects extensive optimization across target biology, linker–payload chemistry, dosing, and in vivo pharmacology. In contrast, AM6 is positioned in this study as an exploratory delivery vehicle used to interrogate internalization pathways and intracellular ADC design principles in a controlled in vitro setting. The data are not intended to suggest competitive performance with clinically established targets, but rather to inform early-stage considerations relevant to antibody selection and ADC engineering.

All mechanistic studies were performed in a single TNBC cell line (MDA-MB-231-LM2), and therefore, the observed internalization behavior may not generalize across tumor types or HSPG2 expression contexts. Antigen-negative or HSPG2-knockdown controls were not included, limiting our ability to definitively ascribe ADC uptake and cytotoxicity to HSPG2-dependent processes rather than cell-line-specific or antigen-independent internalization mechanisms. Internalization and trafficking were assessed qualitatively by live-cell confocal microscopy, and quantitative kinetics or absolute uptake measurements were not determined. Finally, all findings are restricted to in vitro evaluation; in vivo pharmacokinetics, biodistribution, toxicity, and antitumor efficacy were not examined and will be essential to assess translational relevance. Accordingly, this study is intended to provide mechanistic insight and comparative guidance for linker/payload selection rather than definitive target validation or therapeutic efficacy claims.

## Data Availability

The original contributions presented in this study are included in the article/Supplementary Material. Further inquiries can be directed to the corresponding author.

## Supplementary Materials

The following supporting information can be downloaded at: https://www.mdpi.com/article/10.3390/cancers18101638/s1, Figure S1: a–e: LC-MS confirmation of the synthesized doxorubicin payloads; Figure S2: AM6 and Ctx internalization movies; Figure S3: MTS assays following treatment with individual endocytic inhibitors; Figure S4: Effect of endocytic inhibitors on individual endocytic marker; Figure S5: HIC analysis of antibody-doxorubicin conjugates; Figure S6: SEC analysis of antibody-doxorubicin conjugates; Table S1: 590nm fluorescence of dox-linkers per μM in 25 μg/mL water solution; Figure S7: Doxorubicin IC50 in MDA-MB-231-LM2 cells; Figure S8: PLRP-qTOF of Ctx-vc-MMAE conjugates; Figure S9: Cytotoxicity of different MMAE ADCs in HSPG2^+^, EGFR^−^ cell lines MB49 and T24; Figure S10: SDS-PAGE for the characterization of Ctx-dox aggregation.
