# Supplementary material for: In Vitro Characterization of Internalization Pathways and Cytotoxic Activity of Anti-HSPG2 Antibody–Drug Conjugates in MDA-MB-231-LM2 Cells
Source: Cancers (Basel). 2026 May 19;18(10):1638. doi: 10.3390/cancers18101638 (PMC13204521; doi:10.3390/cancers18101638)
Supplement: Supplementary file 1 [file cancers-18-01638-s001.zip › cancers-4285520-supplementary.pdf]

## Supplementary information

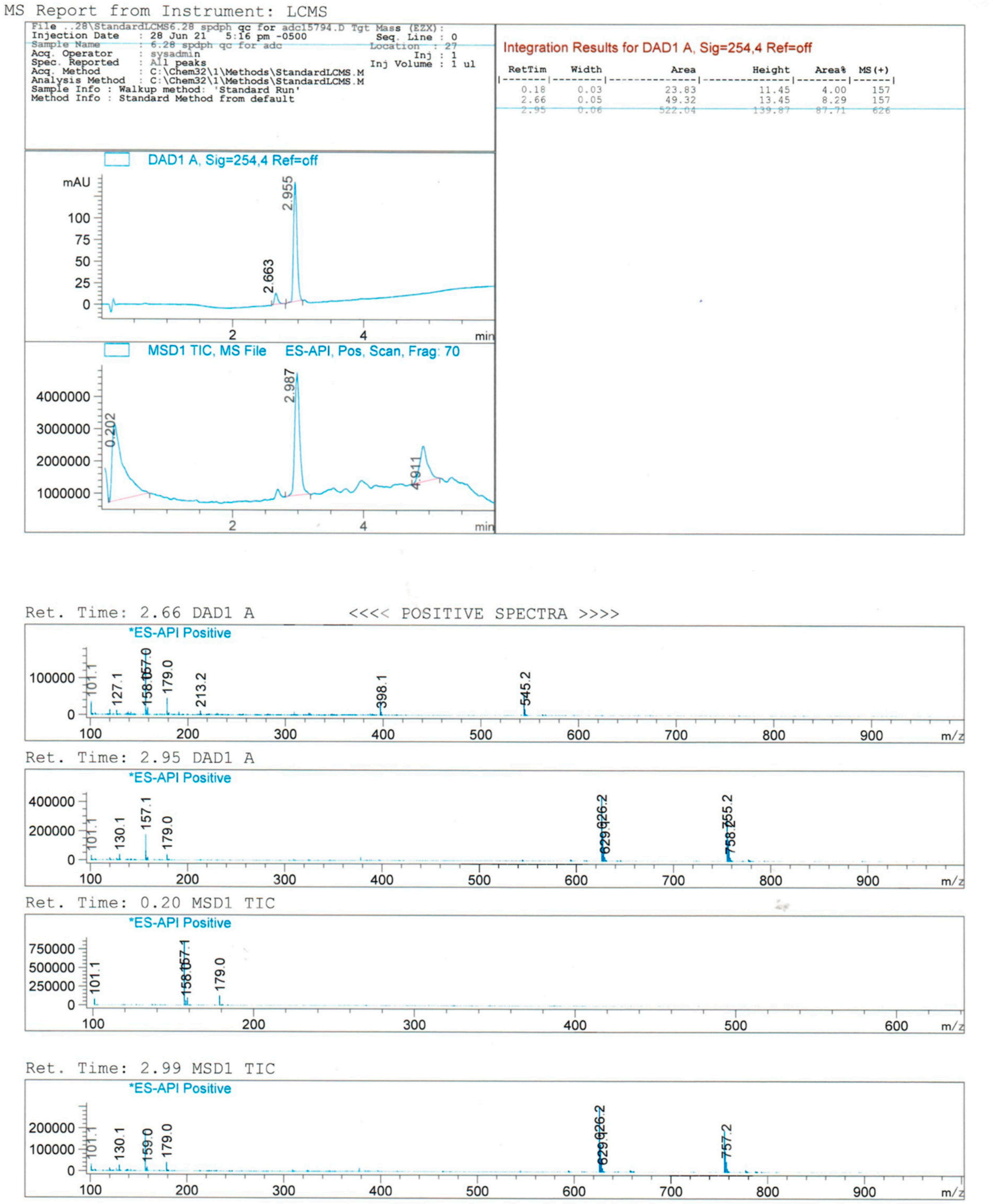


a. LC-MS confirmation of PDPH-dox. $[M+H]^+$=757.2

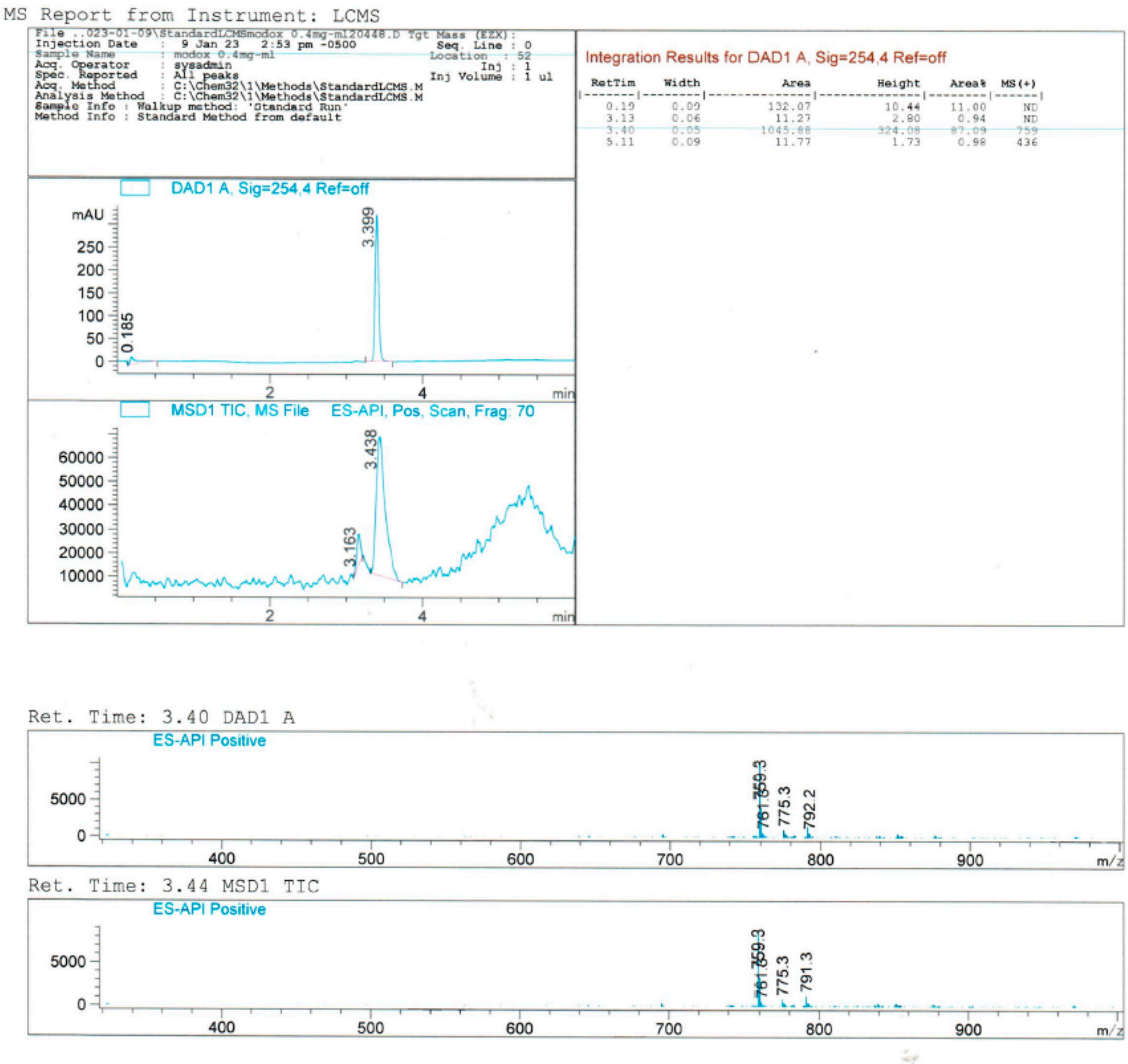

MS Report from Instrument: LCMS

File ..023-01-09\StandardLCMSmcdox 0.4mg-ml20448.D Tgt Mass (EZX):
Injection Date : 9 Jan 23 2:53 pm -0500 Seq. Line : 0
Sample Name : mcdox 0.4mg-ml Location : 52
Acq. Operator : sysadmin Inj : 1
Spec. Reported : All peaks Inj Volume : 1 ul
Acq. Method : C:\Chem32\1\Methods\StandardLCMS.M
Analysis Method : C:\Chem32\1\Methods\StandardLCMS.M
Sample Info : Walkup method: 'Standard Run'
Method Info : Standard Method from default

Integration Results for DAD1 A, Sig=254,4 Ref=off

| RetTim | Width | Area | Height | Area% | MS(+) |
|---|---|---|---|---|---|
| 0.19 | 0.09 | 132.07 | 10.44 | 11.00 | ND |
| 3.13 | 0.06 | 11.27 | 2.80 | 0.94 | ND |
| 3.40 | 0.05 | 1045.88 | 324.08 | 87.09 | 759 |
| 5.11 | 0.09 | 11.77 | 1.73 | 0.98 | 436 |



b. LC-MS confirmation of Mc-dox. $[M+Na]^+$=759.2

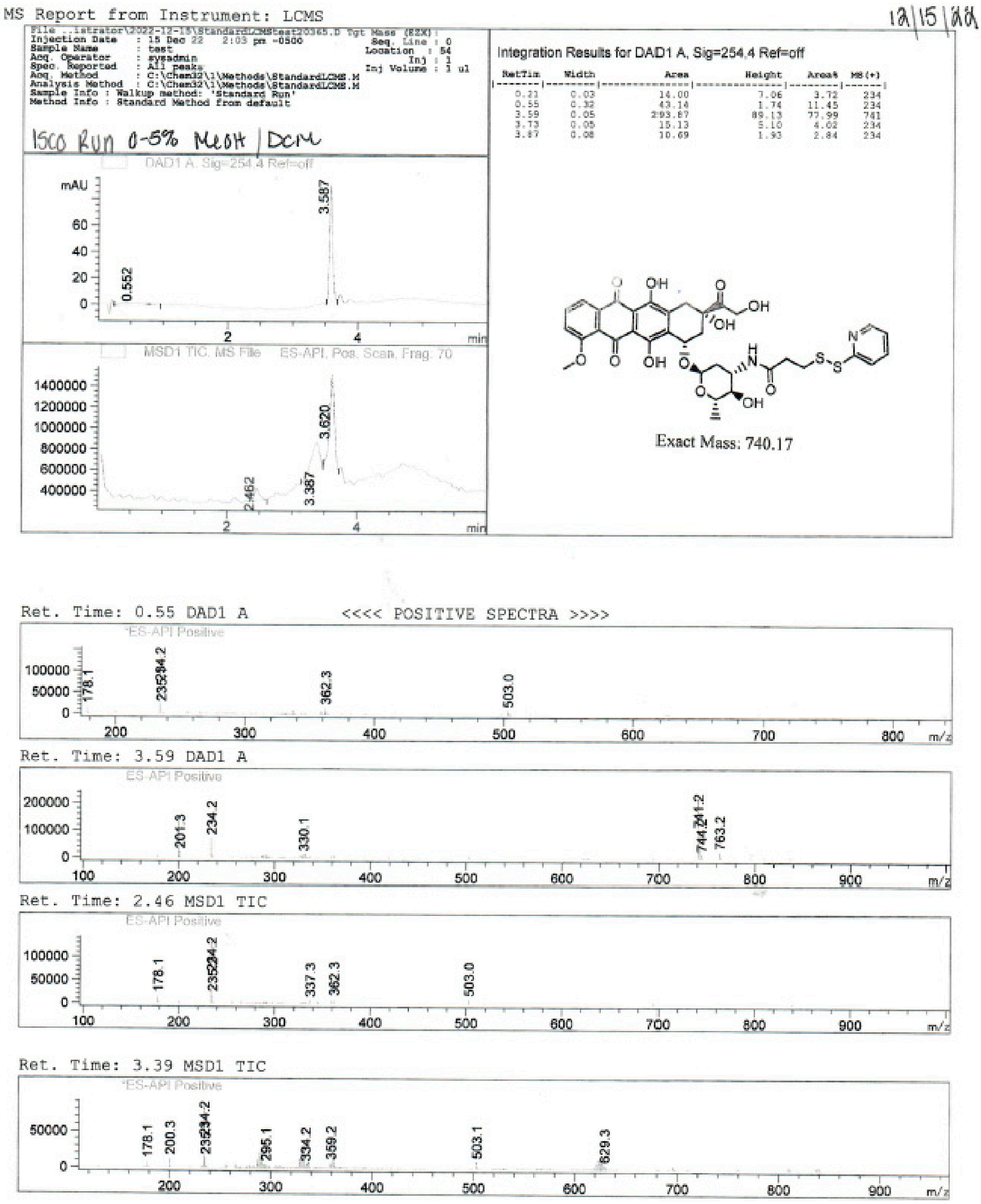

MS Report from Instrument: LCMS

12/15/22

```
File ..istrator\2022-12-15\StandardLCMStest20365.D Tgt Mass (EZX):
Injection Date     : 15 Dec 22   2:03 pm -0500         Seq. Line : 0
Sample Name        : test                              Location  : 54
Acq. Operator      : sysadmin                                Inj : 1
Spec. Reported     : All peaks                        Inj Volume : 1 ul
Acq. Method        : C:\Chem32\1\Methods\StandardLCMS.M
Analysis Method    : C:\Chem32\1\Methods\StandardLCMS.M
Sample Info : Walkup method: 'Standard Run'
Method Info : Standard Method from default
```

ISCO Run 0-5% MeOH | DCM

Integration Results for DAD1 A, Sig=254.4 Ref=off

| RetTim | Width | Area | Height | Area% | MS(+) |
|---|---|---|---|---|---|
| 0.21 | 0.03 | 14.00 | 7.06 | 3.72 | 234 |
| 0.55 | 0.32 | 43.14 | 1.74 | 11.45 | 234 |
| 3.59 | 0.05 | 293.87 | 89.13 | 77.99 | 741 |
| 3.73 | 0.05 | 15.13 | 5.10 | 4.02 | 234 |
| 3.87 | 0.08 | 10.69 | 1.93 | 2.84 | 234 |



c. LC-MS confirmation of PDP-dox. $[M+H]^+$=741.2

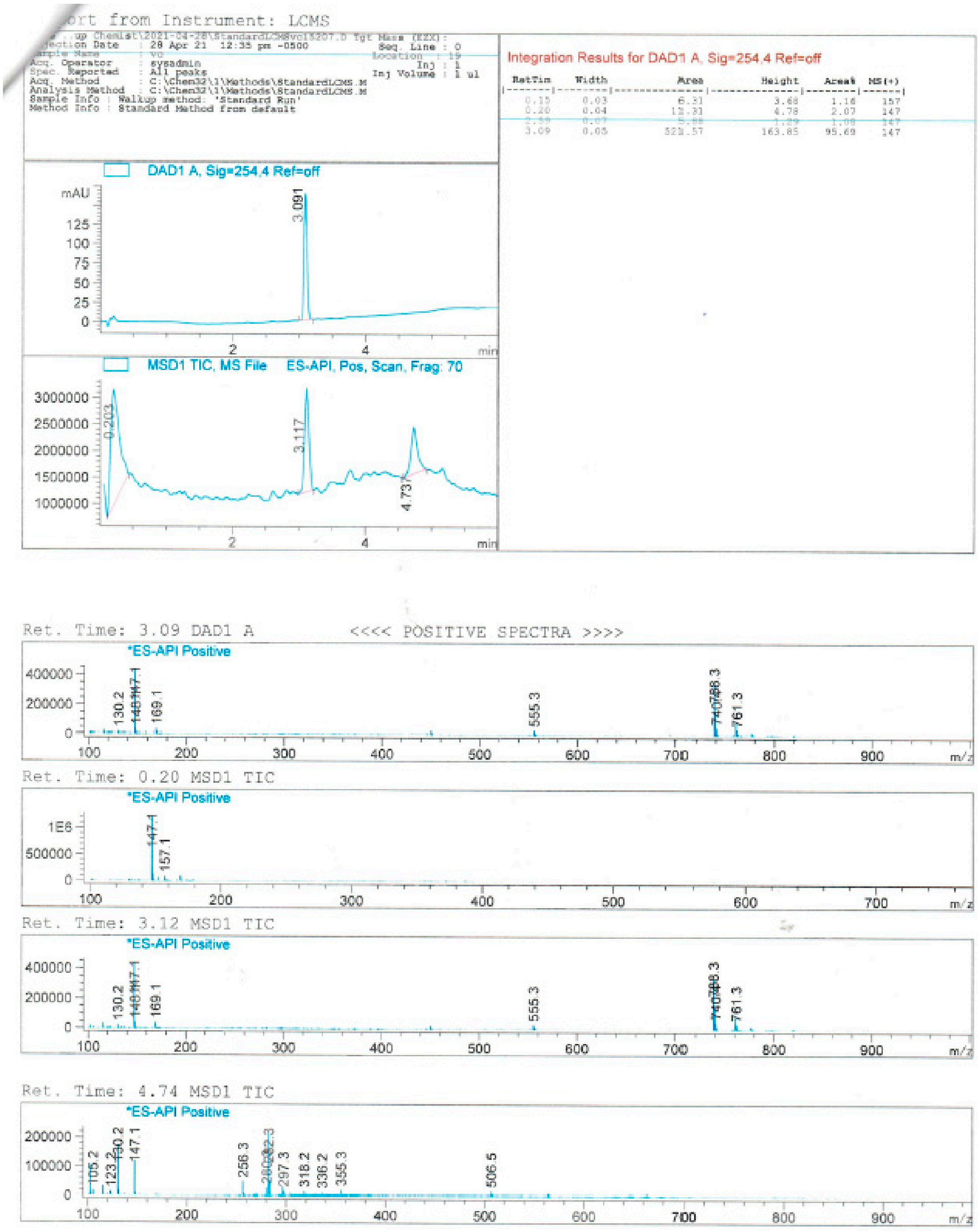


d. LC-MS of Mc-val-cit-dox, $[M + Na]^+$=1164.71, full mass signal reported in e)

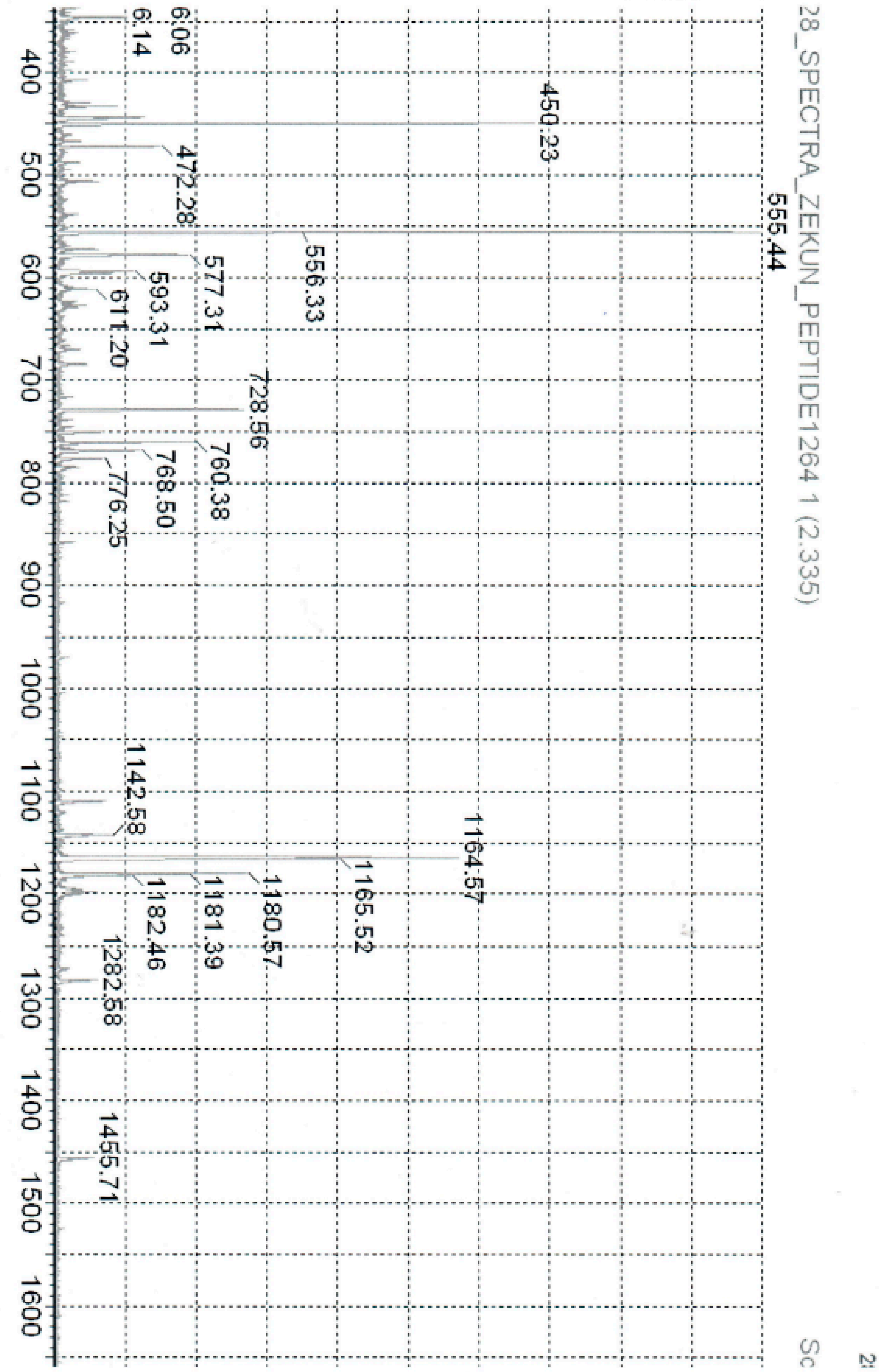


e. Mass report of Mc-val-cit-dox. $[M + Na]^+$=1164.71

Figure S1a-e: MS confirmation of PDPH-dox, mc-dox, PDP-dox, mc-val-cit-dox

Figure S2 (See slides): AM6 and Ctx internalization movies are attached in the separate ppt file

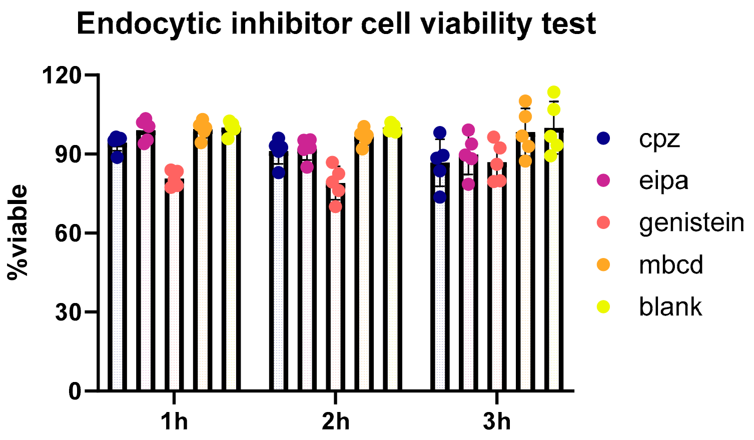


Figure S3: Effect of individual endocytic inhibitors on LM2 cell viability determined using MTS assay, n=5 for each group. The concentration of each inhibitor solution was the same as that used in imaging studies.

Figure S4 (See slides): Effect of endocytic inhibitors on individual endocytic markers are attached in the separate ppt file

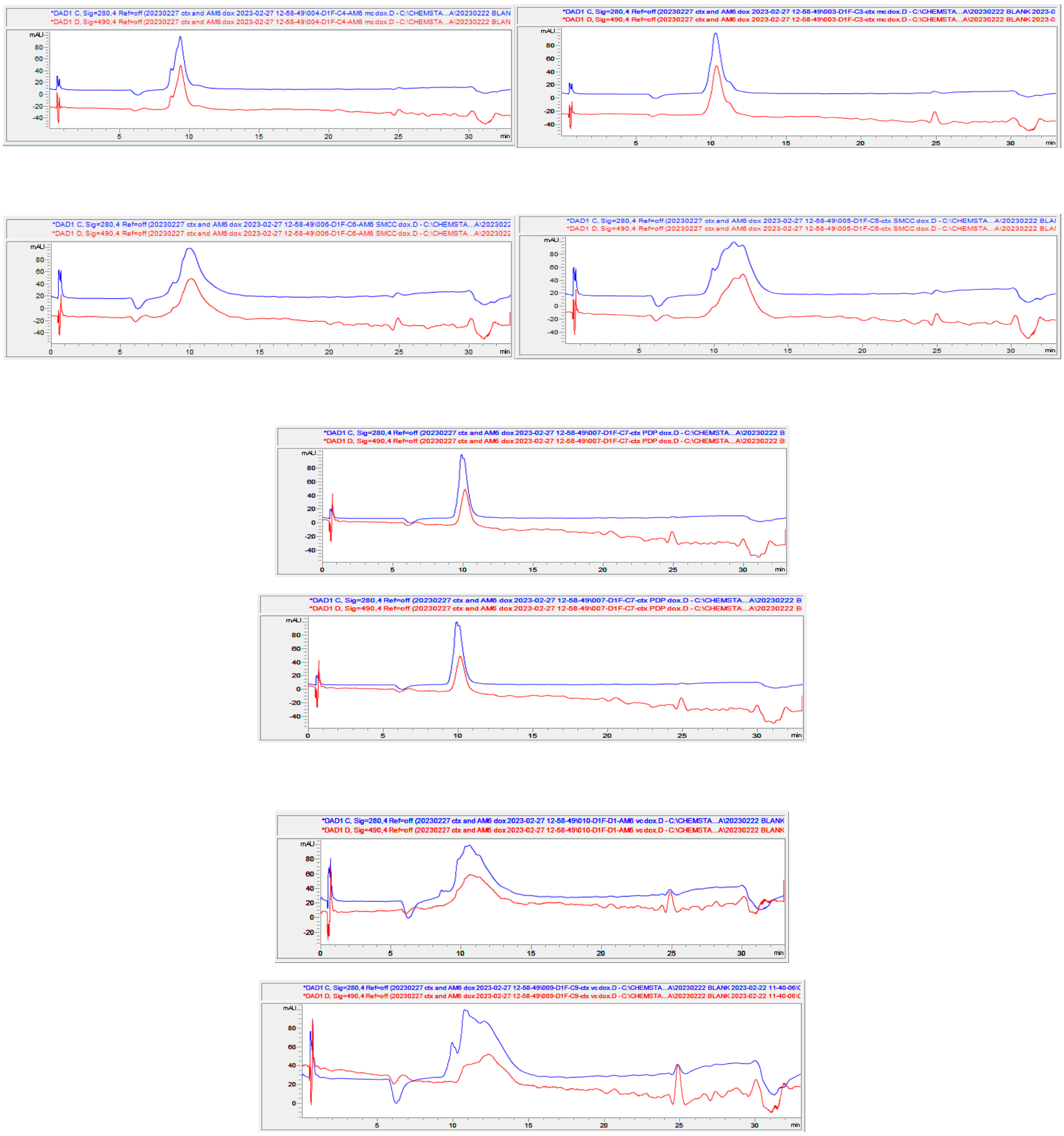

Figure S5: HIC analysis of antibody-doxorubicin conjugates. Left column chromatograms: AM6 conjugates; Right column chromatograms: Ctx conjugates. Dox linker from top to bottom: PDPH-dox, mc-dox, SMCC-dox, PDP-dox, vc-dox.

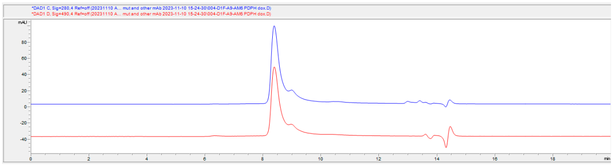
*DAD1 C, Sig=280,4 Ref=off (20231110 A... mut and other mAb 2023-11-10 15-24-30\004-D1F-A9-AM6 PDPH dox.D)
*DAD1 D, Sig=490,4 Ref=off (20231110 A... mut and other mAb 2023-11-10 15-24-30\004-D1F-A9-AM6 PDPH dox.D)
mAU
min

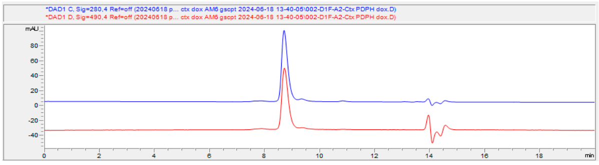
*DAD1 C, Sig=280,4 Ref=off (20240618 p... ctx dox AM6 gscpt 2024-06-18 13-40-05\002-D1F-A2-Ctx PDPH dox.D)
*DAD1 D, Sig=490,4 Ref=off (20240618 p... ctx dox AM6 gscpt 2024-06-18 13-40-05\002-D1F-A2-Ctx PDPH dox.D)
mAU
min

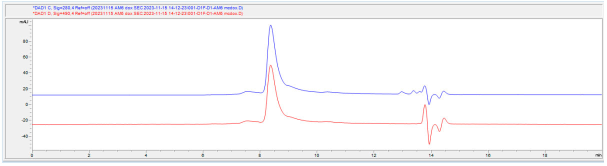
*DAD1 C, Sig=280,4 Ref=off (20231115 AM6 dox SEC 2023-11-15 14-12-23\001-D1F-D1-AM6 mcdox.D)
*DAD1 D, Sig=490,4 Ref=off (20231115 AM6 dox SEC 2023-11-15 14-12-23\001-D1F-D1-AM6 mcdox.D)
mAU
min

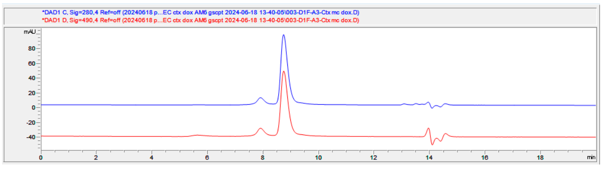
*DAD1 C, Sig=280,4 Ref=off (20240618 p...EC ctx dox AM6 gscpt 2024-06-18 13-40-05\003-D1F-A3-Ctx mc dox.D)
*DAD1 D, Sig=490,4 Ref=off (20240618 p...EC ctx dox AM6 gscpt 2024-06-18 13-40-05\003-D1F-A3-Ctx mc dox.D)
mAU
min

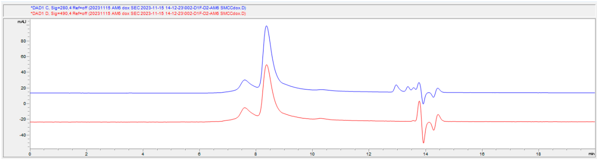
*DAD1 C, Sig=280,4 Ref=off (20231115 AM6 dox SEC 2023-11-15 14-12-23\002-D1F-D2-AM6 SMCCdox.D)
*DAD1 D, Sig=490,4 Ref=off (20231115 AM6 dox SEC 2023-11-15 14-12-23\002-D1F-D2-AM6 SMCCdox.D)
mAU
min

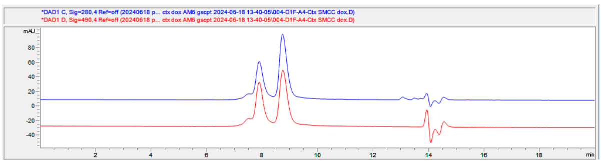
*DAD1 C, Sig=280,4 Ref=off (20240618 p... ctx dox AM6 gscpt 2024-06-18 13-40-05\004-D1F-A4-Ctx SMCC dox.D)
*DAD1 D, Sig=490,4 Ref=off (20240618 p... ctx dox AM6 gscpt 2024-06-18 13-40-05\004-D1F-A4-Ctx SMCC dox.D)
mAU
min

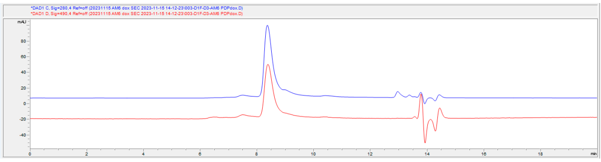
*DAD1 C, Sig=280,4 Ref=off (20231115 AM6 dox SEC 2023-11-15 14-12-23\003-D1F-D3-AM6 PDPdox.D)
*DAD1 D, Sig=490,4 Ref=off (20231115 AM6 dox SEC 2023-11-15 14-12-23\003-D1F-D3-AM6 PDPdox.D)
mAU
min

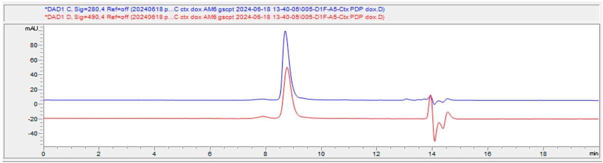
*DAD1 C, Sig=280,4 Ref=off (20240618 p...C ctx dox AM6 gscpt 2024-06-18 13-40-05\005-D1F-A5-Ctx PDP dox.D)
*DAD1 D, Sig=490,4 Ref=off (20240618 p...C ctx dox AM6 gscpt 2024-06-18 13-40-05\005-D1F-A5-Ctx PDP dox.D)
mAU
min

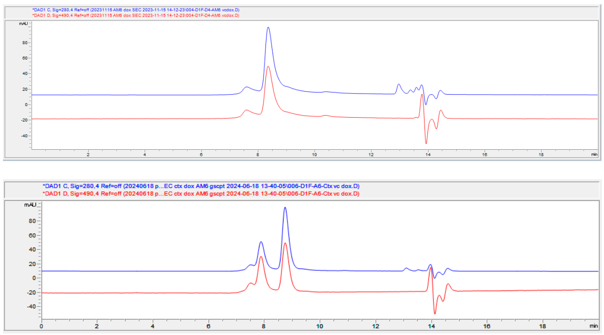


Figure S6: SEC analysis of antibody-doxorubicin conjugates. Left column chromatograms: AM6 conjugates; Right column chromatograms: Ctx conjugates. Dox linker from top to bottom: PDPH-dox, mc-dox, SMCC-dox, PDP-dox, vc-dox.

| Linker-dox | Fluor intensity/μM |
| --- | --- |
| PDPH | 4.71e4 |
| Mc | 5.14e4 |
| SMCC | 4.18e4 |
| PDP | 3.25e4 |
| Vc | 1.30e4 |

Table S1. 590 nm fluorescence of dox-linkers per μM in 25 μg/ml water solution. Ex = 490 nm.

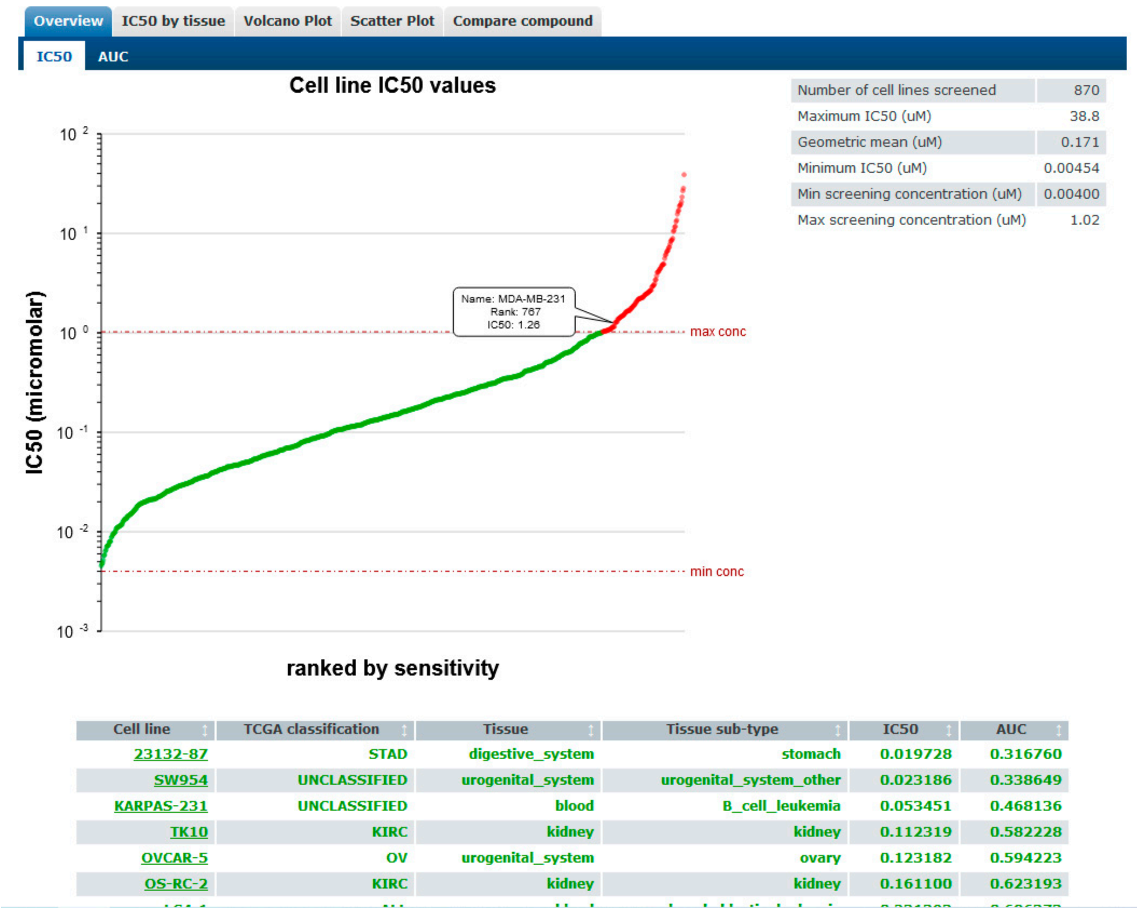


| Cell line | TCGA classification | Tissue | Tissue sub-type | IC50 | AUC |
|---|---|---|---|---|---|
| 23132-87 | STAD | digestive_system | stomach | 0.019728 | 0.316760 |
| SW954 | UNCLASSIFIED | urogenital_system | urogenital_system_other | 0.023186 | 0.338649 |
| KARPAS-231 | UNCLASSIFIED | blood | B_cell_leukemia | 0.053451 | 0.468136 |
| TK10 | KIRC | kidney | kidney | 0.112319 | 0.582228 |
| OVCAR-5 | OV | urogenital_system | ovary | 0.123182 | 0.594223 |
| OS-RC-2 | KIRC | kidney | kidney | 0.161100 | 0.623193 |

Figure S7: Doxorubicin IC50 on MDA-MB-231 from GDSC datasets. Doxorubicin IC50 on LM2 cells was approximately 1.26 µM

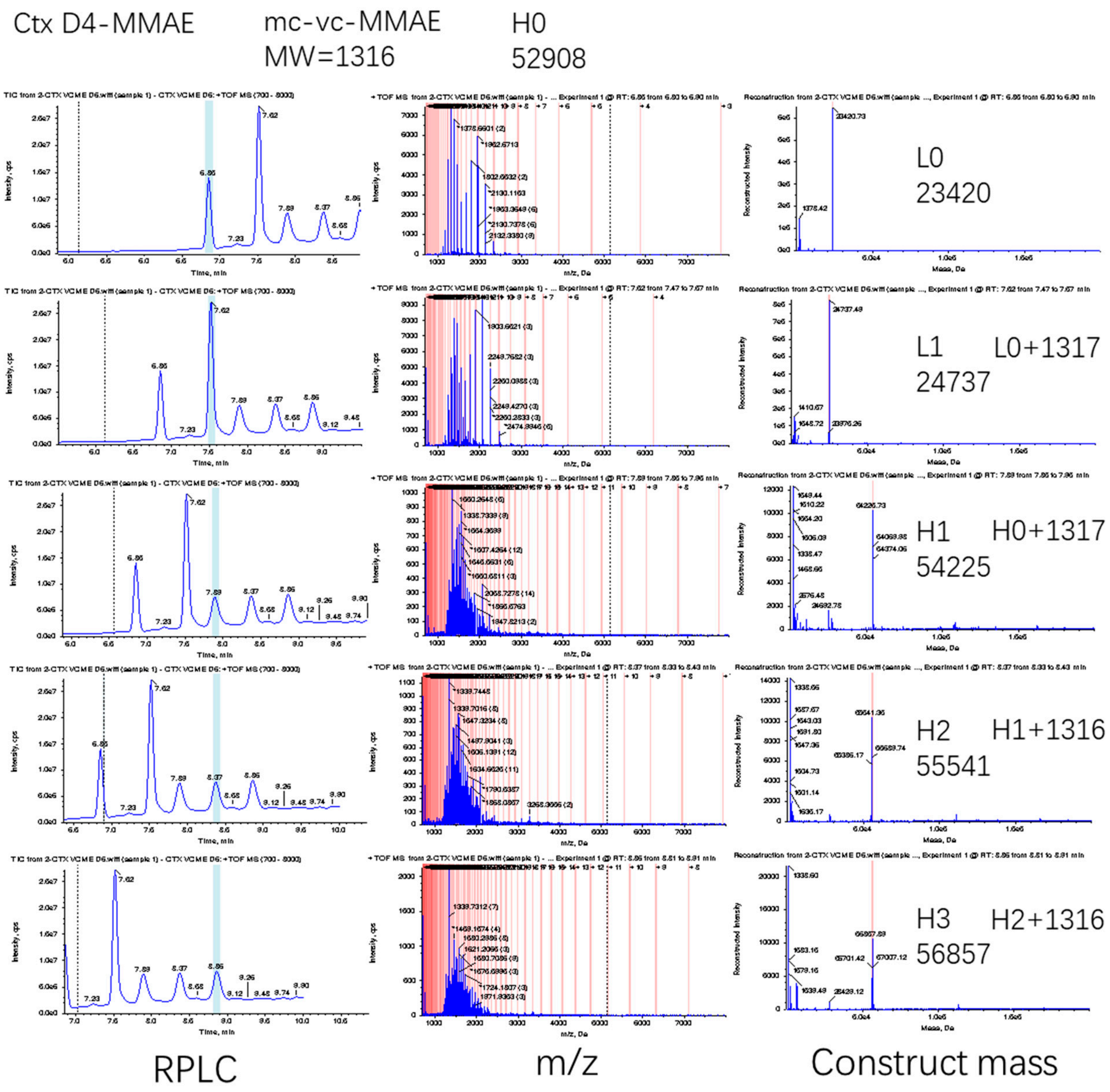


Figure S8: PLRP-qTOF of ctx-vcMMAE conjugates

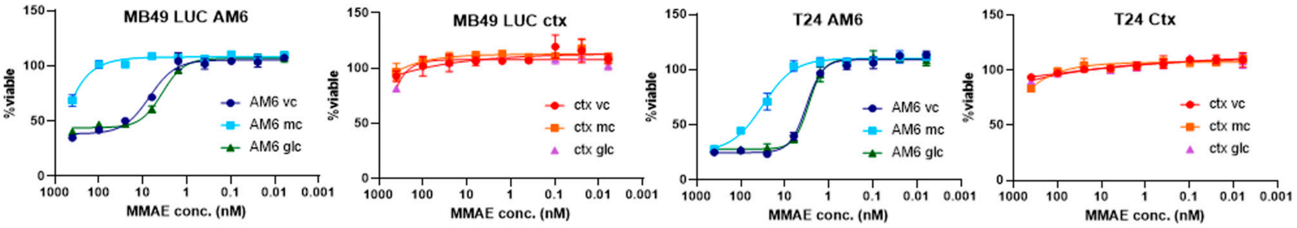


Figure S9: Cytotoxicity of different MMAE ADCs in HSPG2+, EGFR- cell lines MB49 and T24

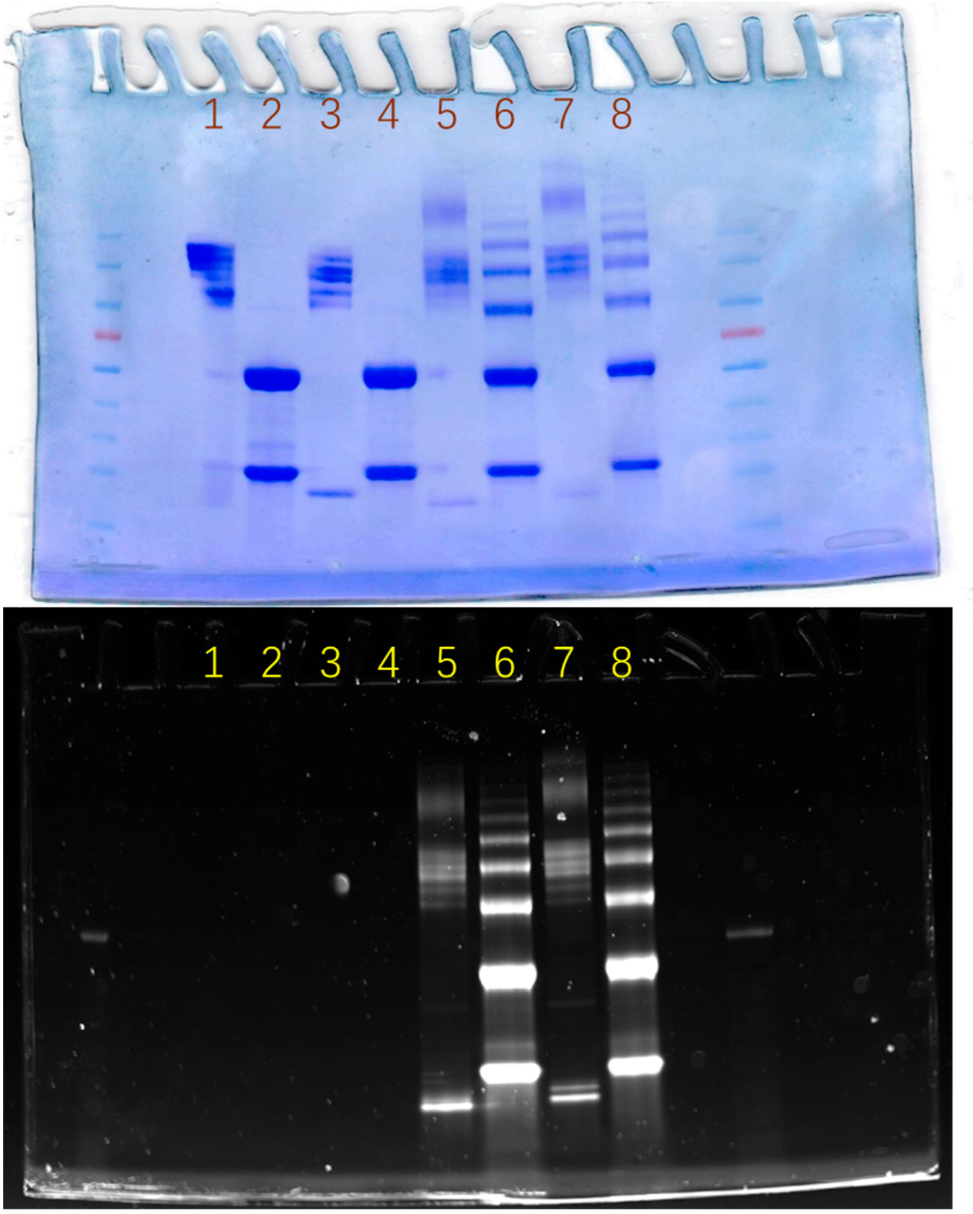


Figure S10: SDS-PAGE for the characterization of Ctx-dox aggregation. The same gel was imaged with the fluorescent gel imager to identify doxorubicin-related bands (bottom), then stained with Coomassie blue to identify protein bands (top). Native sample Lane 1: Ctx; Lane 3: Ctx-vcMMAE; Lane 5: Ctx SMCC-dox; Lane 7: Ctx vc-dox. Each sample was also analyzed under fully denaturing conditions (50 mM DTT, 90 °C, 30mins) in Lanes 2/4/6/8. Diffusion of DTT leads to the partial reduction/ denaturation of the lane 1/3/5/7
