# Supplementary material for: In Vitro Characterization of Internalization Pathways and Cytotoxic Activity of Anti-HSPG2 Antibody–Drug Conjugates in MDA-MB-231-LM2 Cells
Source: Cancers (Basel). 2026 May 19;18(10):1638. doi: 10.3390/cancers18101638 (PMC13204521; doi:10.3390/cancers18101638)
Supplement: Supplementary file 1 [file cancers-18-01638-s001.zip › Supp Fig in slides.pptx]

## Slide 1
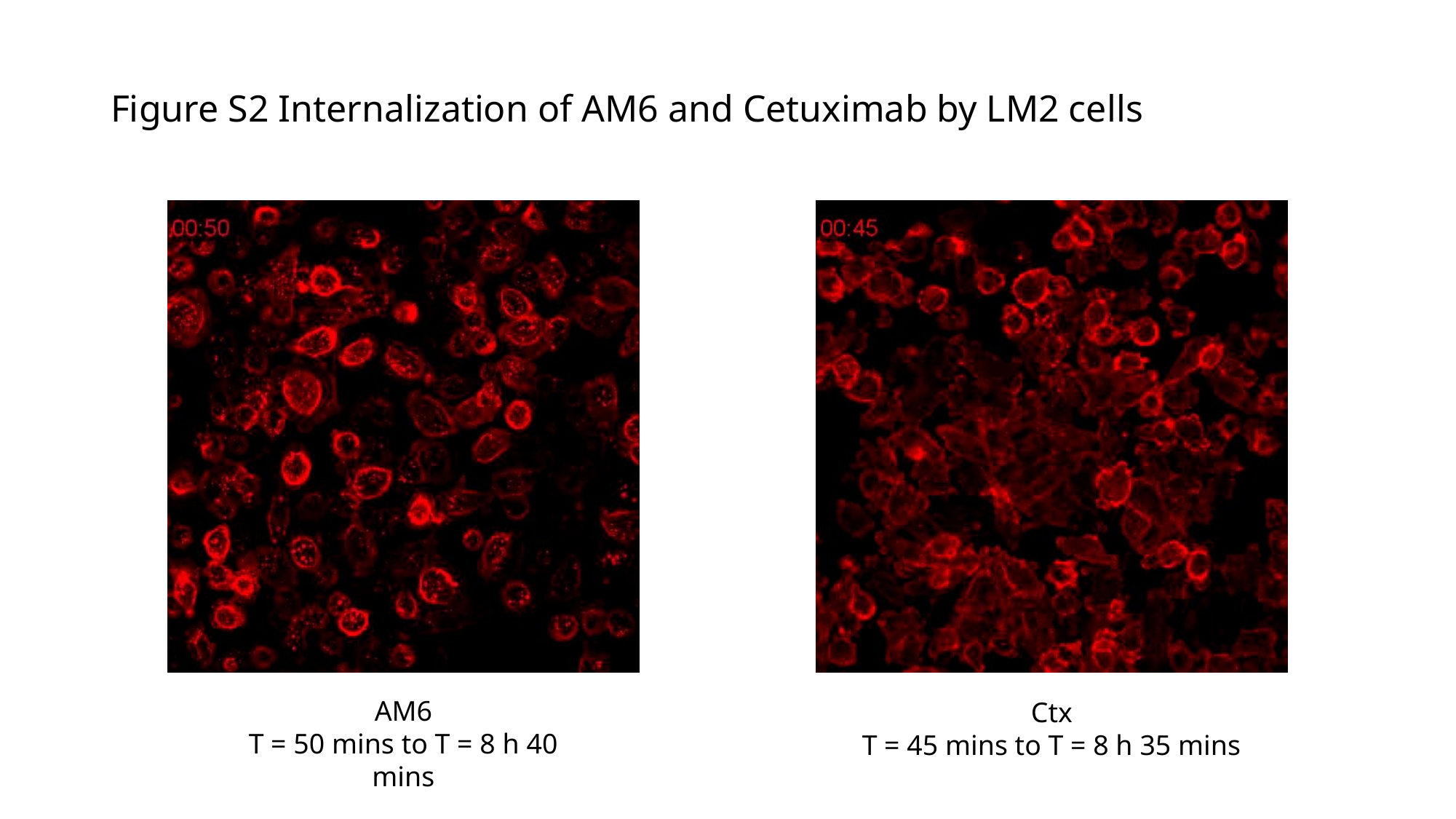

Figure S2 Internalization of AM6 and Cetuximab by LM2 cells
AM6
T = 50 mins to T = 8 h 40 mins
Ctx
T = 45 mins to T = 8 h 35 mins

## Slide 2
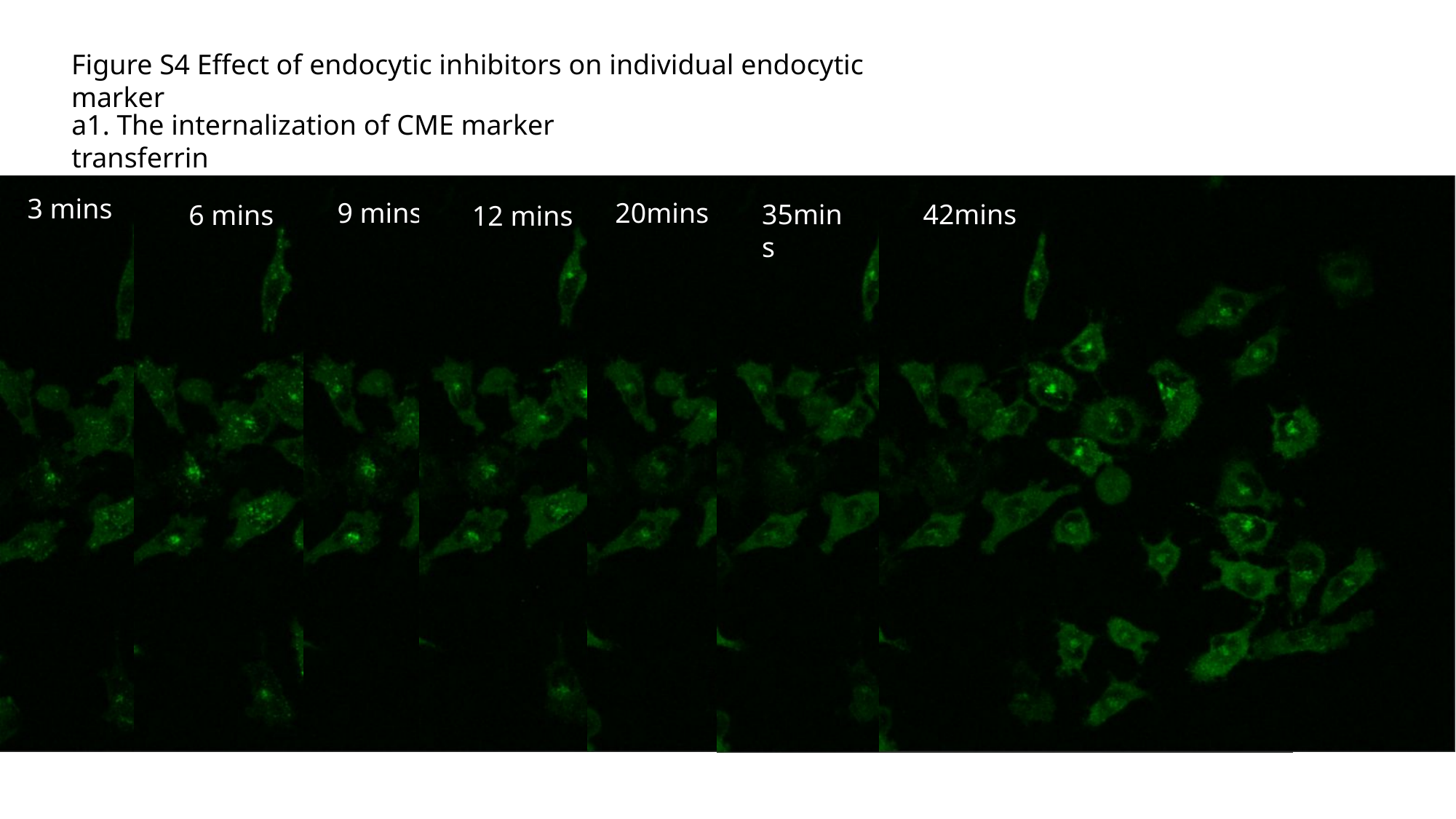

Figure S4 Effect of endocytic inhibitors on individual endocytic marker
a1. The internalization of CME marker transferrin
42mins
20mins
33 mins
6 mins
9 mins
12 mins
35mins

## Slide 3
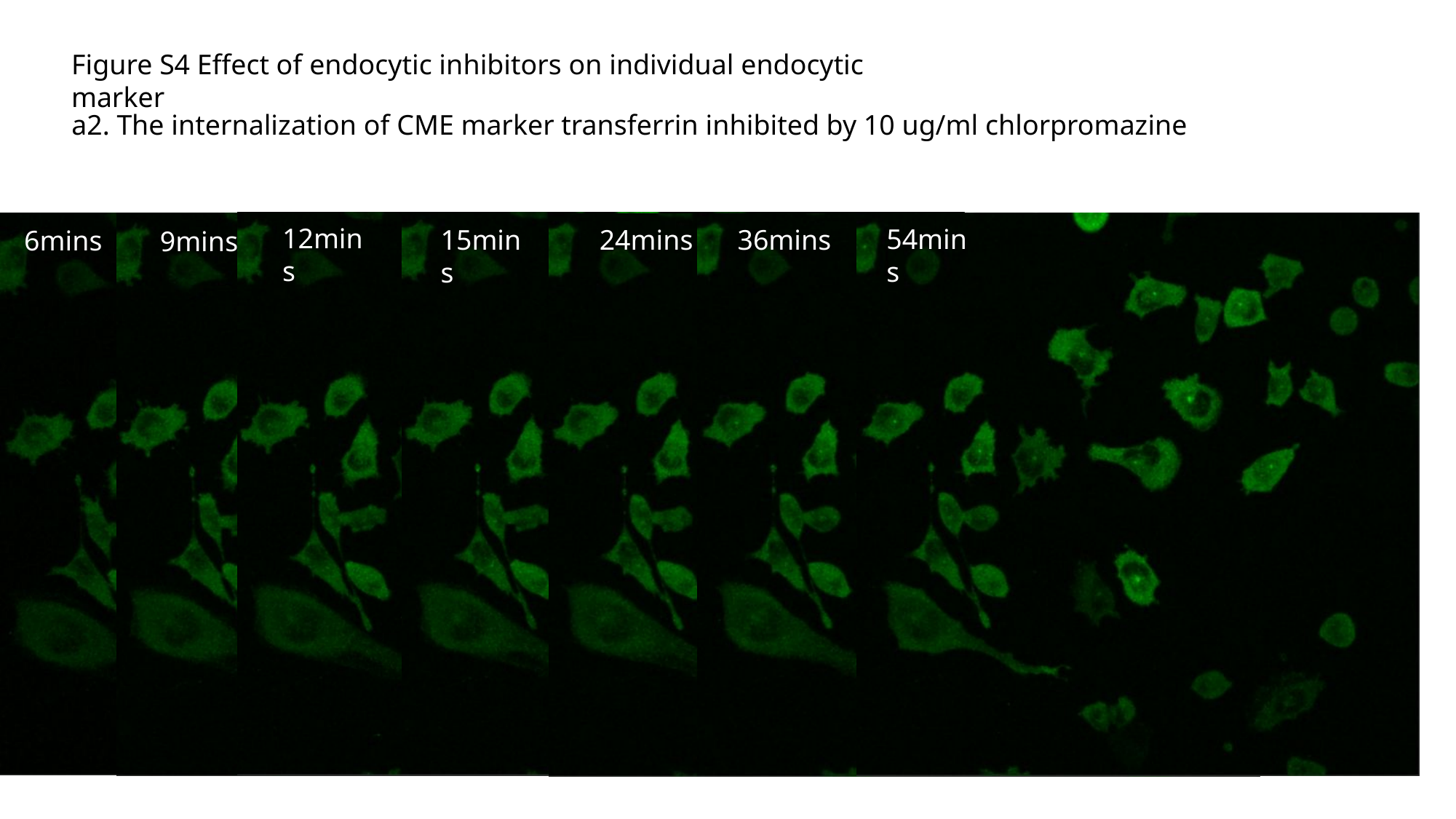

Figure S4 Effect of endocytic inhibitors on individual endocytic marker
a2. The internalization of CME marker transferrin inhibited by 10 ug/ml chlorpromazine
12mins
15mins
9mins
6mins
54mins
24mins
36mins

## Slide 4
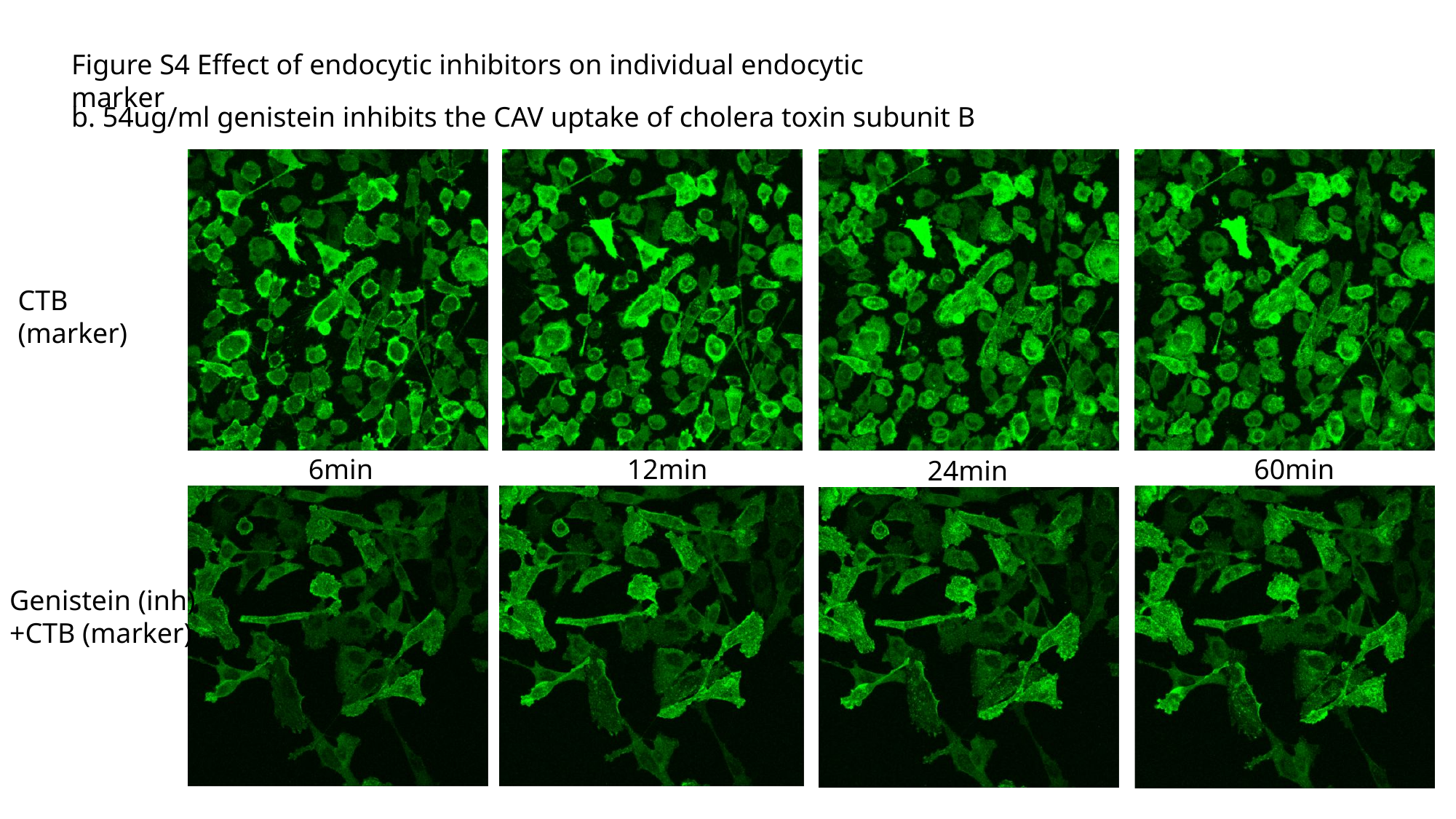

Figure S4 Effect of endocytic inhibitors on individual endocytic marker
b. 54ug/ml genistein inhibits the CAV uptake of cholera toxin subunit B
CTB (marker)
60min
6min
12min
24min
Genistein (inh)
+CTB (marker)

## Slide 5
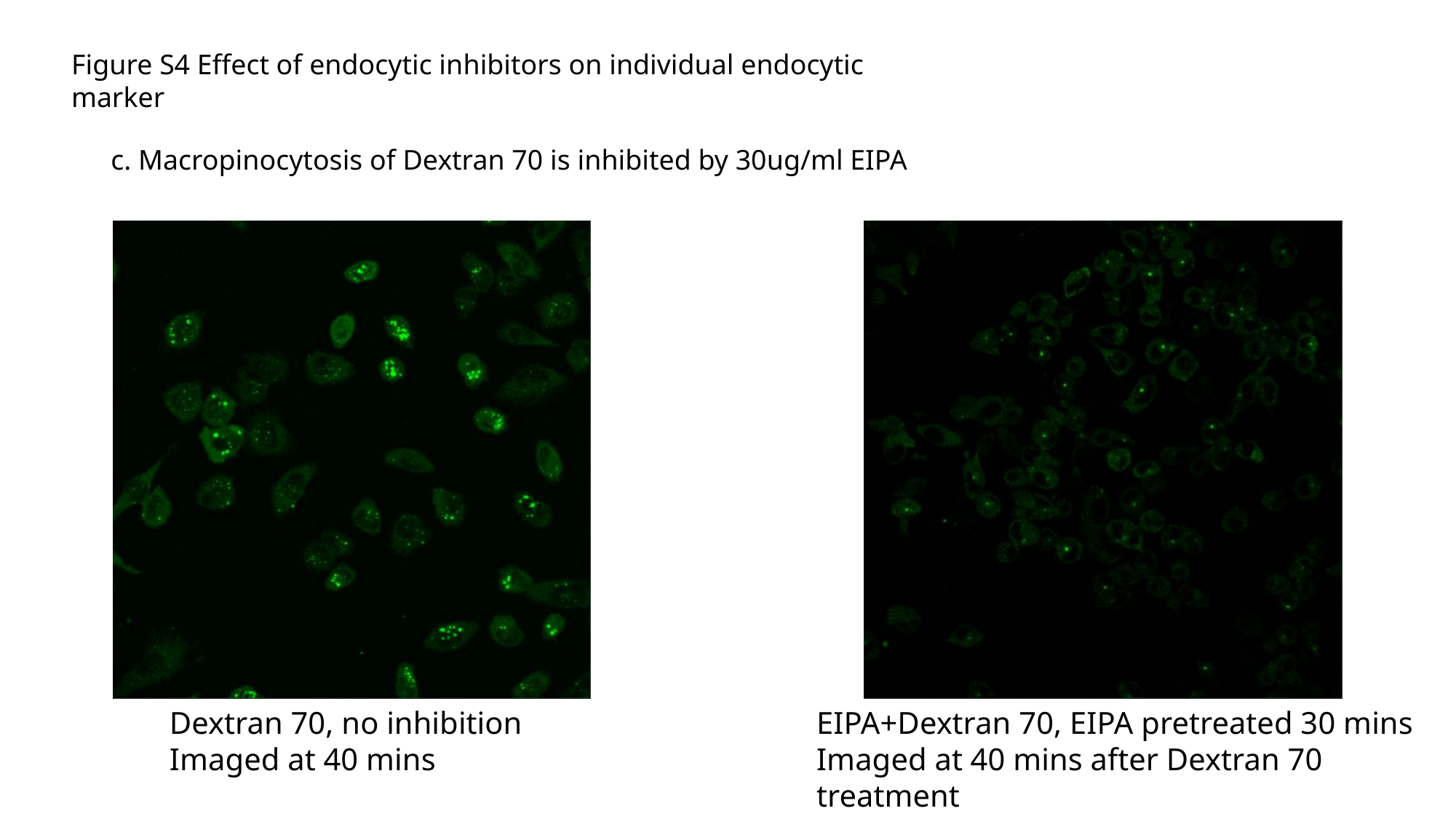

Figure S4 Effect of endocytic inhibitors on individual endocytic marker
c. Macropinocytosis of Dextran 70 is inhibited by 30ug/ml EIPA
Dextran 70, no inhibition
Imaged at 40 mins
EIPA+Dextran 70, EIPA pretreated 30 mins
Imaged at 40 mins after Dextran 70 treatment
